# JCD-DEA: a joint covariate detection tool for differential expression analysis on tumor expression profiles

**DOI:** 10.1186/s12859-019-2893-3

**Published:** 2019-06-28

**Authors:** Yi Li, Yanan Liu, Yiming Wu, Xudong Zhao

**Affiliations:** College of Information and Computer Engineering, Northeast Forestry University, No.26 Hexing Road, Harbin, 150040 China

**Keywords:** Feature selection, Expression profiles, Differential expression analysis, Diagnosis, Cancer

## Abstract

**Background:**

Differential expression analysis on tumor expression profiles has always been a key issue for subsequent biological experimental validation. It is important how to select features which best discriminate between different groups of patients. Despite the emergence of multivariate analysis approaches, prevailing feature selection methods primarily focus on multiple hypothesis testing on individual variables, and then combine them for an explanatory result. Besides, these methods, which are commonly based on hypothesis testing, view classification as a posterior validation of the selected variables.

**Results:**

Based on previously provided A5 feature selection strategy, we develop a joint covariate detection tool for differential expression analysis on tumor expression profiles. This software combines hypothesis testing with testing according to classification results. A model selection approach based on Gaussian mixture model is introduced in for automatic selection of features. Besides, a projection heatmap is proposed for the first time.

**Conclusions:**

Joint covariate detection strengthens the viewpoint for selecting variables which are not only individually but also jointly significant. Experiments on simulation and realistic data show the effectiveness of the developed software, which enhances the reliability of joint covariate detection for differential expression analysis on tumor expression profiles. The software is available at http://bio-nefu.com/resource/jcd-dea.

**Electronic supplementary material:**

The online version of this article (10.1186/s12859-019-2893-3) contains supplementary material, which is available to authorized users.

## Background

Multiple hypothesis testing, which is a situation where more than one hypothesis is evaluated simultaneously [1], has been widely used for differential expression analysis on tumor expression profiles. In order to improve the statistical power, methods that address multiple testing by adjusting the *p*-value from a statistical test have been widely proposed for controlling the family-wise error rate (FWER) [2], false discovery rate (FDR) [3], q-value [4], etc.

Correspondingly, many tools deriving from multiple hypothesis testing have been produced for detecting differentially expressed genes. The *siggenes* bioconductor package, which uses the significance analysis of microarrays (SAM) [5], provides a resampling-based multiple testing procedure involving permutations of data. Linear models for microarray data (namely, *limma*), which help to shrink the estimated sample variances towards an estimate based on all gene variances, provide several common options (e.g., FWER and FDR) for multiple testing [6, 7]. The *multtest* package provides a wide range of resampling-based methods for both FWER and FDR correction [8]. Besides, a regression framework is proposed to estimate the proportion of null hypotheses conditional on observed covariates for controlling FDR [9].

Apart from multiple hypothesis testing on individual variables, multivariate hypothesis testing which indicates whether two distributions of samples are differential or not (e.g., Hotelling’s t^2^-test [10]) holds a non-mainstream position, considering the need of high dimensional matrix operation. With the increasing number of multidimensional features, multiple hypothesis testing also has to be provided to multivariate hypothesis testing, which needs more computation. Therefore, testing according to classification results is assured of a common place. Using classifiers (i.e., logistic regression model, supporting vector machine and random forest, etc. [11]), genes which together help to stratify sample populations are regarded as predictive.

In fact, it has been pointed out that hypothesis testing is regarded to be explanatory, while classification-based methods are viewed to be predictive [12]. As to multiple hypothesis testing on individual variables, it may leave out the explanatory signature. It has been found out in our previous researches [13, 14] that an explanatory pair expressed differently between two patient groups may not be composed of individually explanatory variables. As to various dimensional hypothesis testing and classification-based methods, how to select features not only obeying population distribution but also improving prediction accuracy needs to be further discussed. Thus, we proposed joint covariate detection for differential expression analysis on tumor expression profiles [13]. Three improvements have been made. First of all, we made a bottom-up enumeration of features in different dimensions of gene tuples. Secondly, various dimensional hypothesis testing was combined with classification-based method. Thirdly, a resampling procedure involving permutations of data, which was derived from A5 formulation [15], was constructed. Besides, a combined projection using cancer and adjacent normal tissues was made other than treating them separately [16–19], in order to make a better discriminative performance.

In this paper, we propose a joint covariate detection software for differential expression analysis on tumor expression profiles (i.e., abbreviated to JCD-DEA). In addition, we make three more improvements. Firstly, a model selection method based on Gaussian mixture model (GMM) [20] is introduced in, due to the need of automatic selection of features. Secondly, we present a projection heatmap other than traditional expression heatmap, which directly indicates the effectiveness of JCD-DEA. Thirdly, it is further discussed whether the adjacent normal tissues really work or not.

## Method

**Fig. 1:**
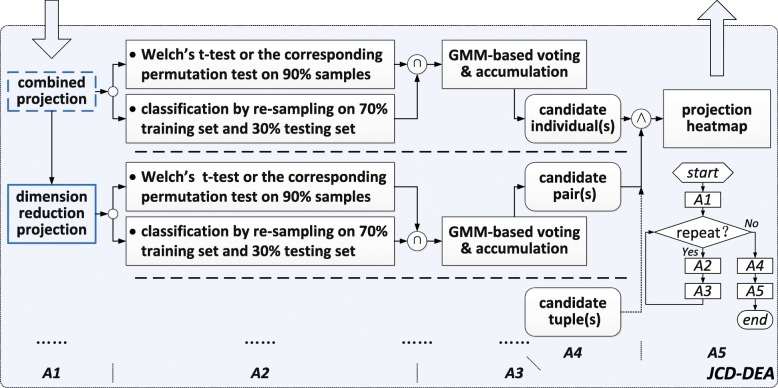
Schematic of JCD-DEA

Our JCD-DEA is concisely expressed, as illustrated in Fig. 1. At step A1, combined projection which corresponds to a linear projection (e.g., Fisher’s linear discriminate analysis [11]) of cancer and adjacent normal tissues on each gene is manually selected or not. Once combined projection is selected, two expression profiles which correspond to cancer and adjacent normal tissues respectively are merged into one projection profiles with two kinds of classification labels (e.g., metastasis or not). Dimension reduction projection refers to a linear projection across genes for enumeration of features in different dimensions bigger than one.

At step A2, values of expressions or projections with two kinds of classification labels are resampled at 90% in each dimension. Welch’s t-test is used on the one dimensional values of two categories for hypothesis testing. Permutations of data are alternatively utilized for overcoming the limitation of sample size. In addition, a classifier is trained using resampled 70% specimens and tested using the left 30% samples. An average classification error rate is calculated after certain rounds of resampling. More details about step A1 and step A2 can be seen in [13].

At step A3, hypothesis testing results are combined with those of classification-based testing. Unlike the voting strategy applied in [13], a GMM-based model selection method [20] for automatic feature selection is introduced in. The numbers of Gaussian mixtures for both *p*-values derived from hypothesis testing and average classification error rates are confirmed respectively. An intersection of features derived from the two minimum-mean-value Gaussian components respectively for hypothesis testing and classification-based testing is obtained and voted with one score for bonus point, as labeled with symbol $\bigcap $ in Fig. 1. As shown in the flow chart of Fig. 1, step A2 and step A3 are repeated for score accumulation in order to ensure the reliability of the selected candidates.

Based on proposed bottom-up enumeration strategy on features with different dimensions, the above procedure is repeated beneath the upper bound of computing capacity. Tuples with different dimensions are voted and accumulated. GMM-based model selection [20] is again used for selection of features in each dimension. The Gaussian component with the minimum-mean-value for accumulation scores is chosen corresponding to candidates. If there is only one Gaussian component in a certain dimension, no candidates in this dimension are to be selected. Considering the discrimination power, candidates are to be chosen with dimensions as high as possible, as labeled with symbol $\bigwedge $ in Fig. 1.

At step A5, we present a projection heatmap other than traditional expression heatmap for further decision. Projection values are derived from the expression values of selected candidates using the same projection method at previous steps. In fact, the thought of using a projection heatmap derives from the procedure of accumulations on classification results. Following the treatment of using projections at step A1 and step A2, it is obvious to use projection values for clustering other than to use simple expression values. The performance of candidates with different dimensions is evaluated by their projection heatmaps. According to Occam’s razor criteria [11], a candidate in a lower dimension while with a good clustering result on its projection heatmap is preferred.

## Implementation

JCD-DEA is written mainly in MATLAB, distributed under GNU GPLv3. Variables which are either individually differential or jointly significant for distinguishing between groups of samples are identified. Due to the lack of adjacent normal tissues in some cancer diseases (e.g., brain cancer), Fisher’s linear discriminative analysis (LDA) other than corresponding bilinear projection [21] is also considered.

Due to the existence of repeating steps in JCD-DEA, we make a two-step implementation: a client part in *Client.zip* for analyzing expression profiles on personal computers or workstations, and a server part in *Server.zip* which is designed to run on cluster servers that using Portable Batch System(PBS) as scheduling program.

**Fig. 2:**
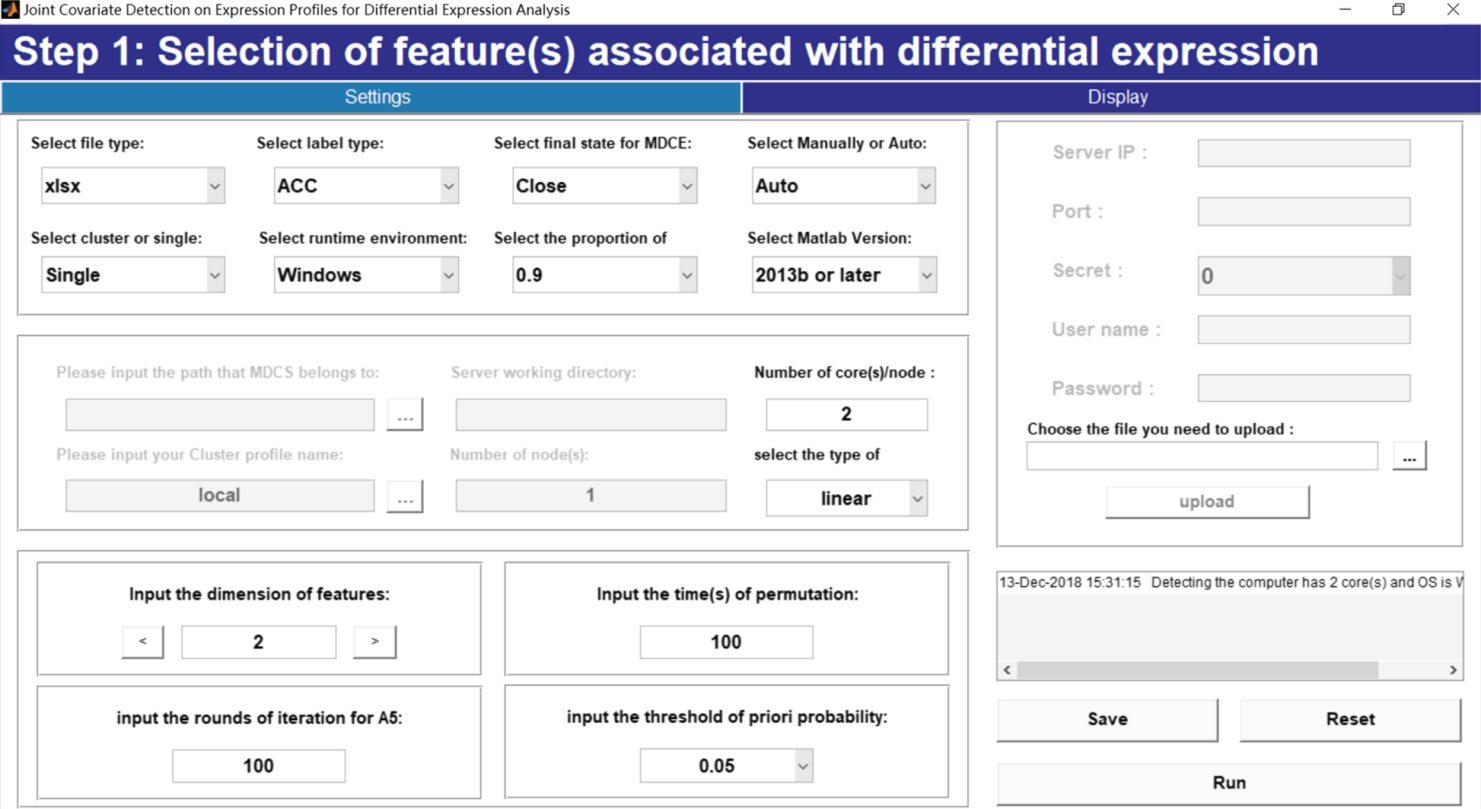
Step 1: Selection of feature(s) associated with differential expression

**Fig. 3:**
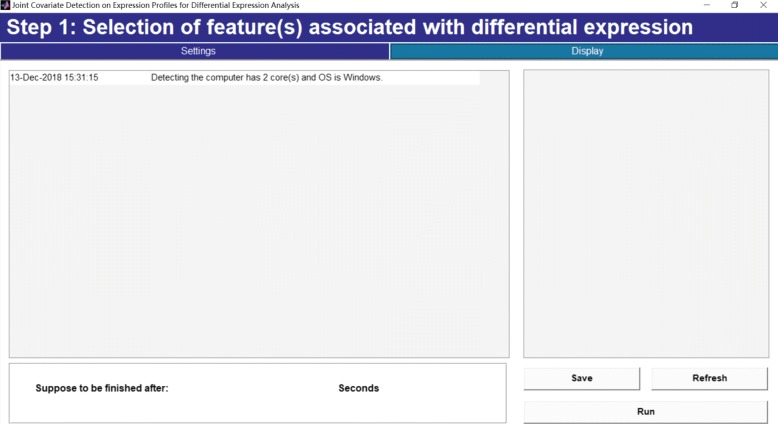
Step 1: Display of computing status

Step A1, step A2 and step A3 correspond to a MATLAB m-file *S1_feature_selection.m* for selection of feature(s) associated with differential expression analysis, as shown in Fig. 2. Parameters for assignment of feature dimension, times of permutation, rounds of iterations for step A2 and step A3, the threshold of prior probability for GMM-based automatic model selection for feature selection and other running environments are set. A display is also made after parameter setting, as shown in Fig. 3.

**Fig. 4:**
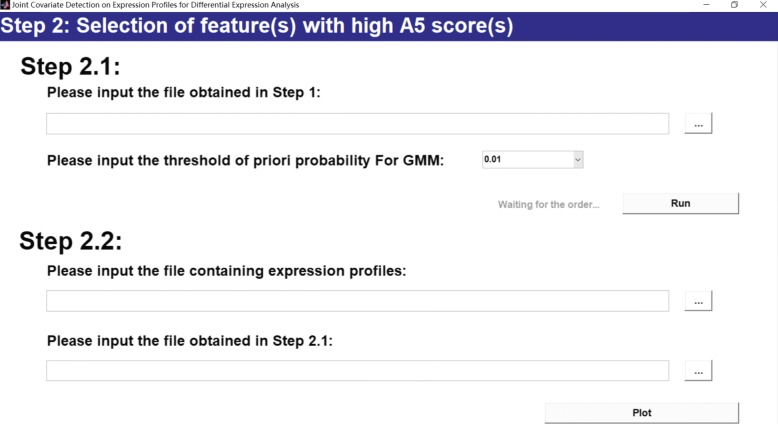
Step 2: Selection of feature(s) with high A5 score(s)

Step A4 and step A5 correspond to a MATLAB m-file *S2_plot_heatmap.m* for selection of feature(s) with high accumulation score(s), as shown in Fig. 4. Candidates derived from step A3 are further selected using GMM-based automatic model selection on their accumulation scores. In addition, a projection heatmap is made for indicating the hierarchical clustering result of each selected feature.

Detailed software documentation and tutorial are presented on http://bio-nefu.com/resource/jcd-dea.

## Results

### Results of the simulated data

In order to exhibit the effectiveness of JCD-DEA, we made a simulated data containing 500 samples equally divided into two categories in a 40 dimensional space. 34 variables of them are independently and identically distributed, each of which keeps a random mean value ranging from 10 to 30 and a same standard deviation 0.01. The left three variable pairs have jointly but not individually significant distributions respectively, subjecting to the following guidelines.

**Fig. 5:**
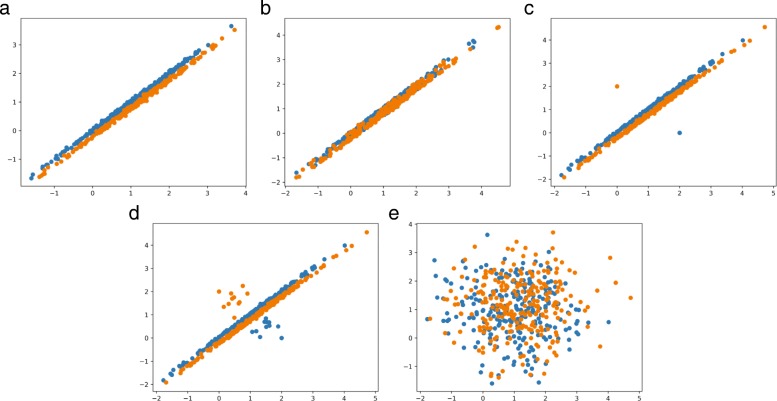
Scatter plots of simulated data in two-dimensional space. **a** The scatter plot with its x-axis and y-axis corresponding to miRNA-alternative 1 and miRNA-alternative 2 **b** The scatter plot with its x-axis and y-axis corresponding to miRNA-alternative 3 and miRNA-alternative 4 **c** The scatter plot with its x-axis and y-axis corresponding to miRNA-alternative 5 and miRNA-alternative 6 **d** An example of unbalanced sampling associated with the scatter plot of c, with undiscovered samples been added **e** The scatter plot with its x-axis and y-axis corresponding to miRNA-alternative 1 and miRNA-alternative 5

As illustrated in Fig. 5a, the variable pair *miRNA-alternative 1* and *miRNA-alternative 2* has a good sample distribution form and also a clear category distinction. The mean vectors corresponding to the two categories of samples are (1,1)^*T*^ and (1.11,0.89)^*T*^. The two categories of samples keep a same covariance matrix, which is expressed as $\left ({\begin {array}{cc} 1&{0.999}\\ {0.999}&1 \end {array}} \right)$.

As to variable pair *miRNA-alternative 3* and *miRNA-alternative 4*, it ought to keep a good sample distribution form but an inferior category distinction. In order to achieve the above objectives, one fifth of samples are randomly and evenly selected and exchanged between the two categories, of which the mean vectors and the covariance matrix keep the same as the former pair before sample exchange, as plotted in Fig. 5b.

Scattered as Fig. 5c, variable pair *miRNA-alternative 5* and *miRNA-alternative 6* appears an inferior sample distribution form but a superior category distinction. Logically speaking, this might be caused by a very small amount of singular points that significantly different from others with the same label. We’ve found this situation in the expression values of miRNA *hsa-mir-450* from data set GSE22058 and make the following surmises for the existence of such points.

- It is just a special case among the expression values of a particular feature, and the corresponding sample should be removed in statistical view.
- This is caused by an unbalanced sampling, which means that there might be undiscovered samples between the singular points and others (see Fig. 5d).

In order to achieve the above objectives, five samples of each category are resampled as singular points with their mean vectors (2,0)^*T*^ and (0,2)^*T*^ and the corresponding covariance matrix $\left ({\begin {array}{cc} 0&{0}\\ {0}&0 \end {array}} \right)$.

Figure 5e shows a scatter plot of *miRNA-alternative 1* and *miRNA-alternative 5*, which illustrates a noncorrelation across different variable pairs.

In fact, we made such a simulated data in order to verify the following three facts.

- Significant feature may not be composed of individual variables expressed differentially between two patient groups.
- Significant feature ought to keep not only a good sample distribution form but also a clear category distinction.
- Projection heatmap corresponding to the classifier selected before may present a better clustering result other than traditional expression heatmap.

Fisher’s LDA was utilized for combined projection and dimension reduction projection at step A1 and the classifier at step A2. Besides, 100 rounds of resampling were performed at step A2 and step A3, with GMM priori probability for eliminating redundant Gaussian components set to 0.001. Correspondingly, GMM priori probability used at step A4 was set to 0.001.

A5 scores (i.e., accumulation scores) together with the *p*-values of Welch’s t-test and the average classification error rate derived from 100 rounds of Fisher’s LDA trained on 70% randomly selected samples and tested on 30% rest samples were calculated. The corresponding pairwise and individual results on simulation data are listed in Tables 1 and 2.

**Table 1:** Individual results on simulation data

miRNA probe	A5 scores	*p*-value	Classification error rate	VIMP using random forests
miRNA-alternative 1	7	0.01774	0.44653	0.00275
miRNA-alternative 2	0	0.90567	0.52247	0.00108
miRNA-alternative 3	0	0.58752	0.51500	0.00043
miRNA-alternative 4	0	0.36873	0.48780	-0.0002
miRNA-alternative 5	2	0.02859	0.47427	0.00174
miRNA-alternative 6	0	0.48969	0.51533	0.00044
miRNA-null 7	0	0.38552	0.51813	-0.00001
miRNA-null 8	14	0.00409	0.44940	0.00139
miRNA-null 9	0	0.16923	0.46687	0.00003
miRNA-null 10	4	0.02509	0.45887	0.00083
miRNA-null 11	0	0.08370	0.47180	0.00080
miRNA-null 12	0	0.68458	0.51887	-0.00011
miRNA-null 13	0	0.82576	0.52187	0.00047
miRNA-null 14	0	0.72355	0.52060	-0.00016
miRNA-null 15	1	0.02793	0.46633	0.00122
miRNA-null 16	0	0.50655	0.51327	0.00002
miRNA-null 17	0	0.58679	0.50447	0.00020
miRNA-null 18	0	0.71515	0.52567	-0.00027
miRNA-null 19	1	0.03970	0.46500	-0.00032
miRNA-null 20	0	0.32140	0.49920	-0.00004
miRNA-null 21	0	0.76909	0.52000	-0.00072
miRNA-null 22	22	0.00030	0.43947	0.00534
miRNA-null 23	0	0.08419	0.46827	0.00086
miRNA-null 24	0	0.15507	0.47913	0.00072
miRNA-null 25	0	0.51227	0.51200	-0.00046
miRNA-null 26	0	0.50874	0.50653	-0.00041
miRNA-null 27	0	0.90546	0.51873	0.00005
miRNA-null 28	0	0.28329	0.47227	-0.00042
miRNA-null 29	0	0.63784	0.50947	-0.00041
miRNA-null 30	0	0.97928	0.52327	-0.00050
miRNA-null 31	0	0.11834	0.48280	0.00063
miRNA-null 32	0	0.91276	0.52140	-0.00044
miRNA-null 33	0	0.08682	0.47747	0.00112
miRNA-null 34	0	0.48329	0.51120	-0.00035
miRNA-null 35	0	0.30921	0.49887	-0.00047
miRNA-null 36	0	0.44131	0.48927	-0.00056
miRNA-null 37	0	0.73472	0.50507	-0.00018
miRNA-null 38	0	0.47165	0.50267	0.00040
miRNA-null 39	0	0.95237	0.51647	-0.00033
miRNA-null 40	0	0.80447	0.52133	0.00018

**Table 2:** Pairwise results on simulation data with a descending order of A5 scores

miRNA probe	miRNA probe	A5 scores	*p*-value	classification error rate
miRNA-alternative 1	miRNA-alternative 2	100	9.4 × 10^−211^	0.00807
miRNA-alternative 5	miRNA-alternative 6	1	7.48 × 10^−8^	0.11633
miRNA-alternative 1	miRNA-alternative 3	0	0.01682	0.45947
...	...	...	...	...
miRNA-alternative 2	miRNA-null 40	0	0.78344	0.53327
miRNA-alternative 3	miRNA-alternative 4	0	4.61 × 10^−45^	0.20433
miRNA-alternative 3	miRNA-alternative 5	0	0.02402	0.47353
...	...	...	...	...
miRNA-null 39	miRNA-null 40	0	0.80111	0.53840

Full results can be seen in Additional file 1: Table S1

In Table 1, it is found that neither A5 scores nor the average classification error rates of individual miRNAs show significance. Several *p*-values (e.g., miRNA-null 8 and miRNA-null 22) exhibit false positives. Besides, variable importance of each miRNA is calculated using random forest [22] as listed in Table 1, which also shows no significance.

In Table 2, it is found that the variable pair *miRNA-alternative 1* and *miRNA-alternative 2* which keeps a statistically good distribution and also a clear category distinction, has the highest A5 score, the minimal *p*-value and the smallest average of classification error rate. As to the variable pair *miRNA-alternative 3* and *miRNA-alternative 4* which keeps a statistically good distribution but an inferior category distinction, a smaller *p*-value and a bigger average of classification error rate are listed. As to the variable pair *miRNA-alternative 5* and *miRNA-alternative 6* which has a statistically inferior distribution but a superior category distinction, it keeps a bigger *p*-value and a smaller average of classification error rate. As the result indicates, only the variable pair *miRNA-alternative 1* and *miRNA-alternative 2* has been selected by JCD-DEA, which shows the effectiveness of our method.

**Fig. 6:**
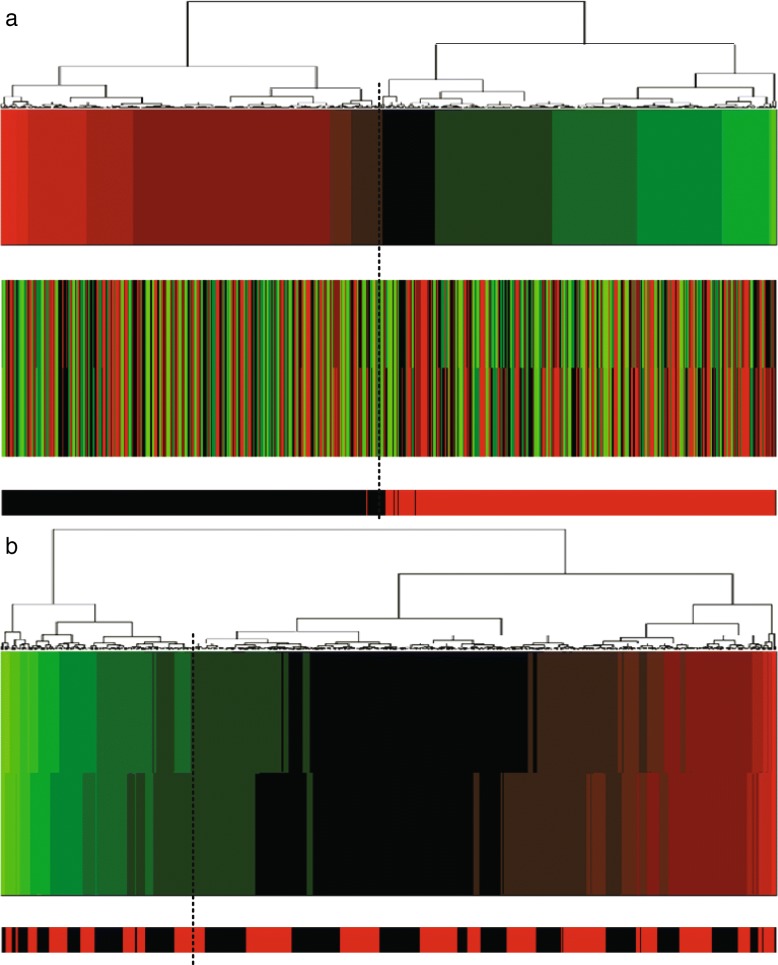
Clustering results of samples using the projection heatmap (up) and the traditional heatmap (down) on miRNA-alternative 1 and miRNA-alternative 2. **a** The result using the projection heatmap **b** The result using the traditional heatmap

**Fig. 7:**
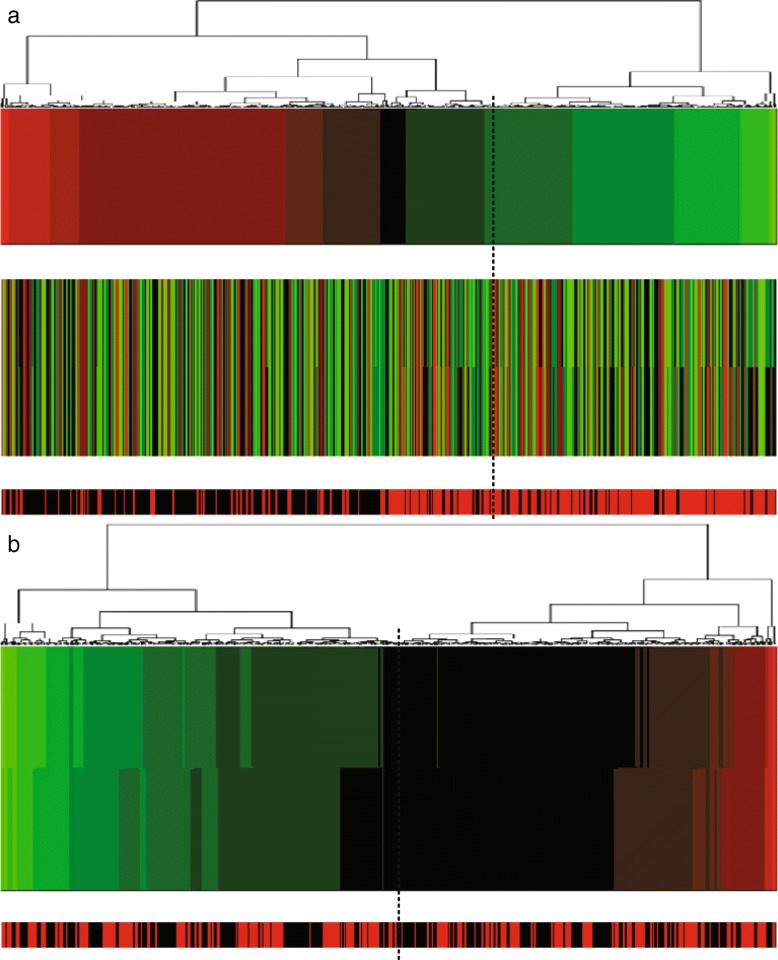
Clustering results of samples using the projection heatmap (up) and the traditional heatmap (down) on miRNA-alternative 3 and miRNA-alternative 4. **a** The result using the projection heatmap **b** The result using the traditional heatmap

**Fig. 8:**
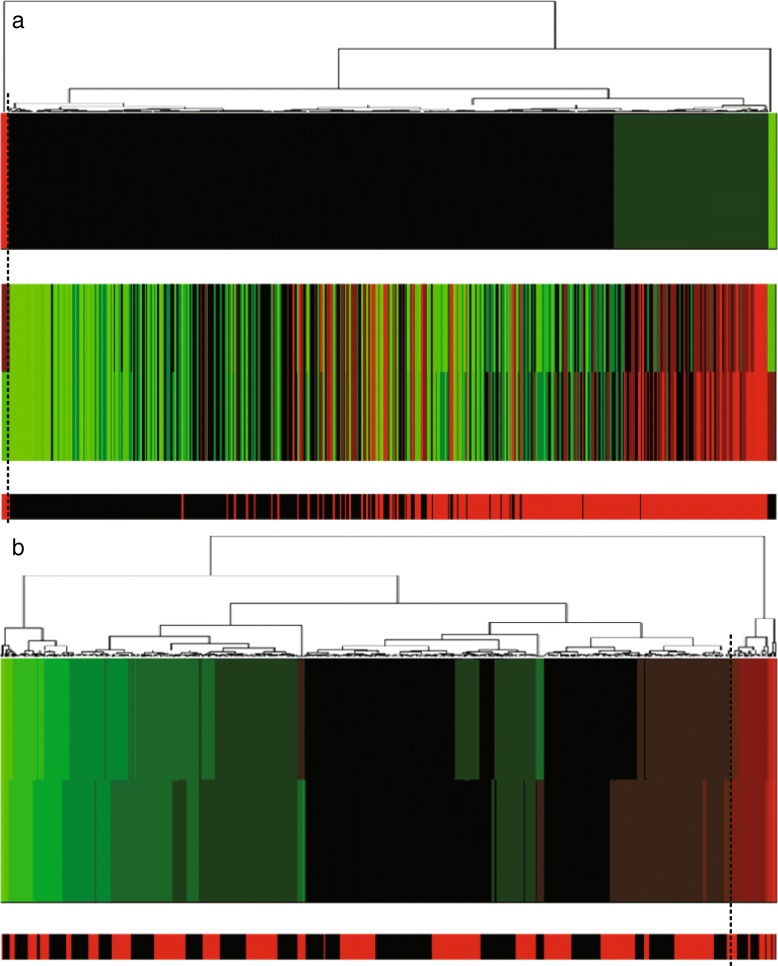
Clustering results of samples using the projection heatmap (up) and the traditional heatmap (down) on miRNA-alternative 5 and miRNA-alternative 6. **a** The result using the projection heatmap **b** The result using the traditional heatmap

In addition, we made projection heatmaps (i.e., clustering on projection values instead of directly on original expression values) as plotted in Figs. 6a, 7a and 8a with the corresponding traditional heatmaps plotted in Figs. 6b, 7b, 8b. In each sub-figure, the up bar, the middle part and the bottom strip refer to the projection values, the expression values and the classification labels, respectively. Slices of the bottom strip colored in red and black in Fig. 6a are clearly separated, compared with Figs. 7a and 8a. Besides, comparisons within each figure show the effectiveness of using a projection heatmap.

### Results of GSE6857

We also performed experiments on GSE6857 which is a public dataset containing 29 samples associated with metastasis cases and 102 samples corresponded to liver cancer without metastasis using linear and bilinear projection. Limited by computing capacity, we have only enumerated features in 2-dimensional space.

Results with GMM priori probability set to 5e-5 are listed in Table 3. Furthermore, only the pair *hsa-mir-29b-1No1* and *hsa-mir-338No1* has been selected with GMM priori probability set to 1e-5.

**Table 3:** A5 voting result on GSE6857 with bilinear projection

miRNA probe	miRNA probe	A5 scores
hsa-mir-29b-1No1	hsa-mir-338No1	409
hsa-mir-210-prec	hsa-mir-30c-2No1	355
hsa-mir-210-prec	hsa-mir-30c-1No1	302
hsa-mir-181b-2No2	hsa-mir-192-2 3No1	282
hsa-mir-031-prec	hsa-mir-215-precNo1	242
hsa-mir-215-precNo2	hsa-mir-371No1	225
hsa-mir-185-precNo1	hsa-mir-194-precNo1	224
hsa-mir-210-prec	hsa-mir-26a-2No1	219
hsa-mir-215-precNo2	hsa-mir-3p21-v3 v4-sense45P	217
hsa-mir-017-precNo1	hsa-mir-210-prec	207
hsa-mir-138-2-prec	hsa-mir-194-precNo1	201
hsa-mir-194-precNo1	hsa-mir-210-prec	196
hsa-mir-138-2-prec	hsa-mir-215-precNo2	191
hsa-mir-210-prec	hsa-mir-215-precNo2	182
hsa-mir-099b-prec-19No1	hsa-mir-124a-2-prec	177
hsa-mir-030b-precNo1	hsa-mir-210-prec	162
hsa-mir-215-precNo1	hsa-mir-338No1	160
hsa-mir-030c-prec	hsa-mir-210-prec	158
hsa-mir-031-prec	hsa-mir-192-2 3No1	157
hsa-mir-135a-2No1	hsa-mir-215-precNo2	153
hsa-mir-191-prec	hsa-mir-210-prec	152
hsa-mir-149-prec	hsa-mir-372No1	149
hsa-mir-105-2No1	hsa-mir-181c-precNo2	145

**Fig. 9:**
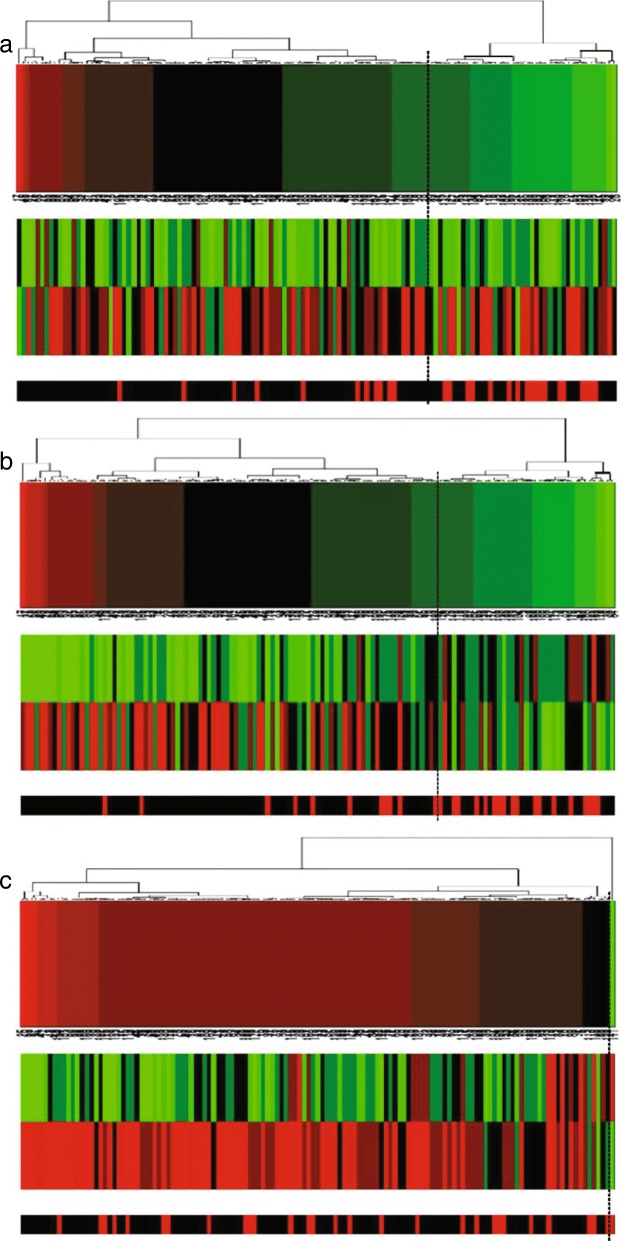
Hierarchical clustering on the selected miRNA pair hsa-mir-29b-1No1 and hsa-mir-338No1. **a** Bilinear projection result **b** Linear projection result on tumor tissues **c** Linear projection result on normal tissues

However, the result is not very ideal. As shown in Fig. 9a, though the red slices of the bottom strip tend to cluster in the right, there are misclassifications. In fact, when diagnosing whether there is metastasis, patients have been diseased. Thus, expressions of normal tissues might not be meaningful anymore.

On account of this, we made new hierarchical clusterings using linear projection on tumor and normal tissues instead of bilinear projection based on the pair selected above respectively. We found that the result on tumor is better than normal tissues, as shown in Fig. 9b and c. The other two pairs pointed in [13] also have the same situation.

**Fig. 10:**
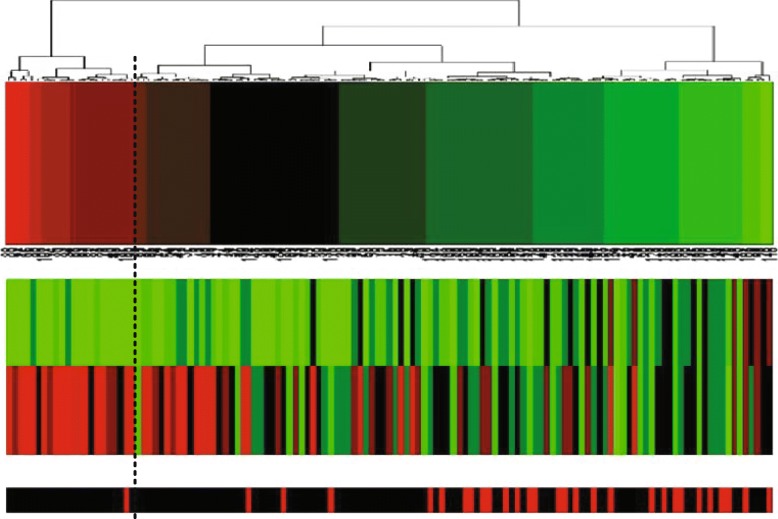
The cluster result of samples using the projection heatmap of the selected feature hsa-mir-194-2No1 and hsa-mir-346No1 on tumor tissues

Thus, we performed new experiments using only linear projection on tumor data with GMM priori probability set to 5e-5. Results are presented in Table 4. And only miRNA pair *hsa-mir-194-2No1* and *hsa-mir-346No1* is selected with GMM priori probability set to 1e-5. Compared to Figs. 9a, 10 indicates that linear projection on tumor tissues have a better clustering result than bilinear projection.

**Table 4:** A5 voting result on GSE6857 with linear projection

miRNA probe	miRNA probe	A5 scores
hsa-mir-194-2No1	hsa-mir-346No1	670
hsa-mir-215-precNo2	hsa-mir-371No1	493
hsa-mir-29b-1No1	hsa-mir-338No1	460
hsa-mir-215-precNo1	hsa-mir-373No2	403
hsa-mir-192-2 3No1	hsa-mir-371No1	401

As illustrated in Fig. 10, the clustering result using projection values of the selected 2-dimension feature can achieve a demonstration effect comparable to the heatmap using expression values on dozen of variables (see Fig. 3 in [13]).

Though improvements have been made in Fig. 10, misclassification still exists, possibly due to the inadequate 2-dimension enumeration limited by our computing capacity.

## Conclusions

JCD-DEA is a bottom-up enumeration tool for seeking not only explanatory but also predictive variables associated with the categories of patients on tumor expression profiles. Other than prevailing differential expression analysis, we concern various dimensional features expressed differentially on tumor expression profiles. In order to strengthen the reliability of selected candidates, both distribution-based and classification-based testing are considered. In addition, we introduce GMM-based model selection for automatic feature selection, which helps to choose features objectively. Finally, a projection heatmap is proposed for hierarchical clustering. On account of the potential possibilities on complicated distributions of samples, we plan to develop new top-down feature selection methods in the near future.

## Availability and requirements

**Project name**: JCD-DEA

**Project home page**: http://bio-nefu.com/resource/jcd-dea

**Operating system(s)**: Linux, Windows

**Programming language**: Matlab (≥R2012b), Python (≥ 3.0)

**License**: GPLv3

**Any restrictions to use by non-academics**: none

## Additional file

Additional file 1 Pairwise results on simulation data with a descending order of A5 scores. (PDF 153 kb)

## Abbreviations

FDR: False discovery rate
FWER: Family-wise error rate
GMM: Gaussian mixture model
JCD-DEA: Joint covariate detection for differential expression analysis
LDA: Fisher’s linear discriminative analysis
PBS: Portable Batch System
SAM: Significance analysis of microarrays
