# Supplementary material for: JCD-DEA: a joint covariate detection tool for differential expression analysis on tumor expression profiles
Source: BMC Bioinformatics. 2019 Jun 28;20:365. doi: 10.1186/s12859-019-2893-3 (PMC6599234; doi:10.1186/s12859-019-2893-3)
Supplement: Supplementary file 1 — Pairwise results on simulation data with a descending order of A5 scores. (PDF 153 kb) [file 12859_2019_2893_MOESM1_ESM.pdf]

miRNA probe	miRNA probe	Row num	Row num	A5 scores	p-value	classification error rates
miRNA-alternative 1	miRNA-alternative 2	1	2	100	9.4E-211	0.008066667
miRNA-alternative 5	miRNA-alternative 6	5	6	1	7.48E-08	0.116333333
miRNA-alternative 1	miRNA-alternative 3	1	3	0	0.016816	0.459466667
miRNA-alternative 1	miRNA-alternative 4	1	4	0	0.008643	0.468533333
miRNA-alternative 1	miRNA-alternative 5	1	5	0	0.001186	0.462133333
miRNA-alternative 1	miRNA-alternative 6	1	6	0	0.012688	0.466133333
miRNA-alternative 1	miRNA-null 7	1	7	0	0.010579	0.447866667
miRNA-alternative 1	miRNA-null 8	1	8	0	0.000288	0.456333333
miRNA-alternative 1	miRNA-null 9	1	9	0	0.006283	0.435266667
miRNA-alternative 1	miRNA-null 10	1	10	0	0.000915	0.447866667
miRNA-alternative 1	miRNA-null 11	1	11	0	0.004324	0.460066667
miRNA-alternative 1	miRNA-null 12	1	12	0	0.017153	0.465333333
miRNA-alternative 1	miRNA-null 13	1	13	0	0.016531	0.468866667
miRNA-alternative 1	miRNA-null 14	1	14	0	0.016298	0.468933333
miRNA-alternative 1	miRNA-null 15	1	15	0	0.000854	0.439733333
miRNA-alternative 1	miRNA-null 16	1	16	0	0.013531	0.465
miRNA-alternative 1	miRNA-null 17	1	17	0	0.015207	0.458866667
miRNA-alternative 1	miRNA-null 18	1	18	0	0.015406	0.459666667
miRNA-alternative 1	miRNA-null 19	1	19	0	0.001929	0.4538
miRNA-alternative 1	miRNA-null 20	1	20	0	0.009641	0.456733333
miRNA-alternative 1	miRNA-null 21	1	21	0	0.016872	0.451333333
miRNA-alternative 1	miRNA-null 22	1	22	0	2.38E-05	0.4162
miRNA-alternative 1	miRNA-null 23	1	23	0	0.002881	0.453466667
miRNA-alternative 1	miRNA-null 24	1	24	0	0.007098	0.452066667
miRNA-alternative 1	miRNA-null 25	1	25	0	0.011972	0.4566
miRNA-alternative 1	miRNA-null 26	1	26	0	0.010429	0.462466667
miRNA-alternative 1	miRNA-null 27	1	27	0	0.017286	0.464066667
miRNA-alternative 1	miRNA-null 28	1	28	0	0.008856	0.457133333
miRNA-alternative 1	miRNA-null 29	1	29	0	0.016287	0.455933333
miRNA-alternative 1	miRNA-null 30	1	30	0	0.017247	0.459866667
miRNA-alternative 1	miRNA-null 31	1	31	0	0.004175	0.446333333
miRNA-alternative 1	miRNA-null 32	1	32	0	0.01738	0.457266667
miRNA-alternative 1	miRNA-null 33	1	33	0	0.003189	0.445466667
miRNA-alternative 1	miRNA-null 34	1	34	0	0.012125	0.468266667
miRNA-alternative 1	miRNA-null 35	1	35	0	0.009231	0.446133333
miRNA-alternative 1	miRNA-null 36	1	36	0	0.012208	0.472866667
miRNA-alternative 1	miRNA-null 37	1	37	0	0.016743	0.459466667
miRNA-alternative 1	miRNA-null 38	1	38	0	0.012756	0.464533333
miRNA-alternative 1	miRNA-null 39	1	39	0	0.017415	0.462
miRNA-alternative 1	miRNA-null 40	1	40	0	0.017159	0.472266667
miRNA-alternative 2	miRNA-alternative 3	2	3	0	0.569023	0.521333333
miRNA-alternative 2	miRNA-alternative 4	2	4	0	0.368445	0.5064
miRNA-alternative 2	miRNA-alternative 5	2	5	0	0.028489	0.489066667
miRNA-alternative 2	miRNA-alternative 6	2	6	0	0.485791	0.519266667
miRNA-alternative 2	miRNA-null 7	2	7	0	0.383327	0.515866667
miRNA-alternative 2	miRNA-null 8	2	8	0	0.003937	0.458666667
miRNA-alternative 2	miRNA-null 9	2	9	0	0.167551	0.490066667
miRNA-alternative 2	miRNA-null 10	2	10	0	0.025074	0.4772
miRNA-alternative 2	miRNA-null 11	2	11	0	0.081311	0.480733333
miRNA-alternative 2	miRNA-null 12	2	12	0	0.665909	0.539
miRNA-alternative 2	miRNA-null 13	2	13	0	0.806932	0.538066667
miRNA-alternative 2	miRNA-null 14	2	14	0	0.710118	0.537

miRNA-alternative 2	miRNA-null 15	2	15	0	0.027922	0.4816
miRNA-alternative 2	miRNA-null 16	2	16	0	0.500991	0.530733333
miRNA-alternative 2	miRNA-null 17	2	17	0	0.577326	0.5186
miRNA-alternative 2	miRNA-null 18	2	18	0	0.705393	0.5286
miRNA-alternative 2	miRNA-null 19	2	19	0	0.039094	0.466
miRNA-alternative 2	miRNA-null 20	2	20	0	0.319106	0.508533333
miRNA-alternative 2	miRNA-null 21	2	21	0	0.751687	0.526866667
miRNA-alternative 2	miRNA-null 22	2	22	0	0.000285	0.4416
miRNA-alternative 2	miRNA-null 23	2	23	0	0.084035	0.473333333
miRNA-alternative 2	miRNA-null 24	2	24	0	0.150894	0.4862
miRNA-alternative 2	miRNA-null 25	2	25	0	0.509902	0.519866667
miRNA-alternative 2	miRNA-null 26	2	26	0	0.508153	0.5186
miRNA-alternative 2	miRNA-null 27	2	27	0	0.869441	0.537066667
miRNA-alternative 2	miRNA-null 28	2	28	0	0.281161	0.497533333
miRNA-alternative 2	miRNA-null 29	2	29	0	0.624357	0.526866667
miRNA-alternative 2	miRNA-null 30	2	30	0	0.904805	0.530333333
miRNA-alternative 2	miRNA-null 31	2	31	0	0.117885	0.489933333
miRNA-alternative 2	miRNA-null 32	2	32	0	0.873833	0.535066667
miRNA-alternative 2	miRNA-null 33	2	33	0	0.086529	0.490333333
miRNA-alternative 2	miRNA-null 34	2	34	0	0.480149	0.524466667
miRNA-alternative 2	miRNA-null 35	2	35	0	0.307364	0.511666667
miRNA-alternative 2	miRNA-null 36	2	36	0	0.43724	0.5198
miRNA-alternative 2	miRNA-null 37	2	37	0	0.718834	0.527066667
miRNA-alternative 2	miRNA-null 38	2	38	0	0.467132	0.517466667
miRNA-alternative 2	miRNA-null 39	2	39	0	0.896523	0.531133333
miRNA-alternative 2	miRNA-null 40	2	40	0	0.783438	0.533266667
miRNA-alternative 3	miRNA-alternative 4	3	4	0	4.61E-45	0.204333333
miRNA-alternative 3	miRNA-alternative 5	3	5	0	0.024022	0.473533333
miRNA-alternative 3	miRNA-alternative 6	3	6	0	0.370136	0.509133333
miRNA-alternative 3	miRNA-null 7	3	7	0	0.306903	0.5048
miRNA-alternative 3	miRNA-null 8	3	8	0	0.003533	0.4604
miRNA-alternative 3	miRNA-null 9	3	9	0	0.134687	0.487533333
miRNA-alternative 3	miRNA-null 10	3	10	0	0.021909	0.476533333
miRNA-alternative 3	miRNA-null 11	3	11	0	0.063538	0.469733333
miRNA-alternative 3	miRNA-null 12	3	12	0	0.494503	0.5296
miRNA-alternative 3	miRNA-null 13	3	13	0	0.555421	0.524
miRNA-alternative 3	miRNA-null 14	3	14	0	0.516121	0.527133333
miRNA-alternative 3	miRNA-null 15	3	15	0	0.022928	0.467333333
miRNA-alternative 3	miRNA-null 16	3	16	0	0.38677	0.524666667
miRNA-alternative 3	miRNA-null 17	3	17	0	0.45301	0.5192
miRNA-alternative 3	miRNA-null 18	3	18	0	0.497695	0.524333333
miRNA-alternative 3	miRNA-null 19	3	19	0	0.031821	0.473266667
miRNA-alternative 3	miRNA-null 20	3	20	0	0.259503	0.517466667
miRNA-alternative 3	miRNA-null 21	3	21	0	0.533139	0.513733333
miRNA-alternative 3	miRNA-null 22	3	22	0	0.00023	0.444066667
miRNA-alternative 3	miRNA-null 23	3	23	0	0.072647	0.4608
miRNA-alternative 3	miRNA-null 24	3	24	0	0.121242	0.498133333
miRNA-alternative 3	miRNA-null 25	3	25	0	0.397041	0.499933333
miRNA-alternative 3	miRNA-null 26	3	26	0	0.404184	0.516066667
miRNA-alternative 3	miRNA-null 27	3	27	0	0.579089	0.521666667
miRNA-alternative 3	miRNA-null 28	3	28	0	0.244791	0.493733333
miRNA-alternative 3	miRNA-null 29	3	29	0	0.48786	0.5126
miRNA-alternative 3	miRNA-null 30	3	30	0	0.586441	0.527933333
miRNA-alternative 3	miRNA-null 31	3	31	0	0.089507	0.479533333

miRNA-alternative 3 miRNA-null 32	3	32	0	0.57583	0.522466667
miRNA-alternative 3 miRNA-null 33	3	33	0	0.075015	0.481
miRNA-alternative 3 miRNA-null 34	3	34	0	0.375785	0.518733333
miRNA-alternative 3 miRNA-null 35	3	35	0	0.2416	0.506933333
miRNA-alternative 3 miRNA-null 36	3	36	0	0.356979	0.519866667
miRNA-alternative 3 miRNA-null 37	3	37	0	0.518326	0.5274
miRNA-alternative 3 miRNA-null 38	3	38	0	0.36038	0.506266667
miRNA-alternative 3 miRNA-null 39	3	39	0	0.586134	0.524933333
miRNA-alternative 3 miRNA-null 40	3	40	0	0.559281	0.5218
miRNA-alternative 4 miRNA-alternative 5	4	5	0	0.018384	0.476266667
miRNA-alternative 4 miRNA-alternative 6	4	6	0	0.265902	0.501466667
miRNA-alternative 4 miRNA-null 7	4	7	0	0.211621	0.518333333
miRNA-alternative 4 miRNA-null 8	4	8	0	0.002564	0.454933333
miRNA-alternative 4 miRNA-null 9	4	9	0	0.10524	0.471333333
miRNA-alternative 4 miRNA-null 10	4	10	0	0.015016	0.442133333
miRNA-alternative 4 miRNA-null 11	4	11	0	0.058322	0.4892
miRNA-alternative 4 miRNA-null 12	4	12	0	0.328288	0.506333333
miRNA-alternative 4 miRNA-null 13	4	13	0	0.357111	0.507466667
miRNA-alternative 4 miRNA-null 14	4	14	0	0.333935	0.504066667
miRNA-alternative 4 miRNA-null 15	4	15	0	0.018336	0.4776
miRNA-alternative 4 miRNA-null 16	4	16	0	0.266759	0.504933333
miRNA-alternative 4 miRNA-null 17	4	17	0	0.283221	0.5076
miRNA-alternative 4 miRNA-null 18	4	18	0	0.343778	0.522733333
miRNA-alternative 4 miRNA-null 19	4	19	0	0.026871	0.4648
miRNA-alternative 4 miRNA-null 20	4	20	0	0.17867	0.496066667
miRNA-alternative 4 miRNA-null 21	4	21	0	0.348274	0.5086
miRNA-alternative 4 miRNA-null 22	4	22	0	0.000225	0.446333333
miRNA-alternative 4 miRNA-null 23	4	23	0	0.048329	0.491333333
miRNA-alternative 4 miRNA-null 24	4	24	0	0.100109	0.4854
miRNA-alternative 4 miRNA-null 25	4	25	0	0.263099	0.478
miRNA-alternative 4 miRNA-null 26	4	26	0	0.252887	0.493066667
miRNA-alternative 4 miRNA-null 27	4	27	0	0.364107	0.513466667
miRNA-alternative 4 miRNA-null 28	4	28	0	0.144187	0.4784
miRNA-alternative 4 miRNA-null 29	4	29	0	0.295312	0.511866667
miRNA-alternative 4 miRNA-null 30	4	30	0	0.368727	0.505466667
miRNA-alternative 4 miRNA-null 31	4	31	0	0.080513	0.491333333
miRNA-alternative 4 miRNA-null 32	4	32	0	0.367539	0.511066667
miRNA-alternative 4 miRNA-null 33	4	33	0	0.050673	0.481533333
miRNA-alternative 4 miRNA-null 34	4	34	0	0.253997	0.508066667
miRNA-alternative 4 miRNA-null 35	4	35	0	0.183585	0.496933333
miRNA-alternative 4 miRNA-null 36	4	36	0	0.227313	0.4862
miRNA-alternative 4 miRNA-null 37	4	37	0	0.339245	0.501466667
miRNA-alternative 4 miRNA-null 38	4	38	0	0.256534	0.502266667
miRNA-alternative 4 miRNA-null 39	4	39	0	0.366461	0.510866667
miRNA-alternative 4 miRNA-null 40	4	40	0	0.344867	0.508066667
miRNA-alternative 5 miRNA-null 7	5	7	0	0.01949	0.461933333
miRNA-alternative 5 miRNA-null 8	5	8	0	0.000422	0.435466667
miRNA-alternative 5 miRNA-null 9	5	9	0	0.008905	0.4742
miRNA-alternative 5 miRNA-null 10	5	10	0	0.00156	0.443533333
miRNA-alternative 5 miRNA-null 11	5	11	0	0.005461	0.4638
miRNA-alternative 5 miRNA-null 12	5	12	0	0.02491	0.483
miRNA-alternative 5 miRNA-null 13	5	13	0	0.028024	0.484466667
miRNA-alternative 5 miRNA-null 14	5	14	0	0.026701	0.483533333
miRNA-alternative 5 miRNA-null 15	5	15	0	0.002099	0.435933333

miRNA-alternative	5	miRNA-null	16	5	16	0	0.019667	0.47
miRNA-alternative	5	miRNA-null	17	5	17	0	0.026755	0.4754
miRNA-alternative	5	miRNA-null	18	5	18	0	0.026094	0.485266667
miRNA-alternative	5	miRNA-null	19	5	19	0	0.003239	0.46
miRNA-alternative	5	miRNA-null	20	5	20	0	0.019011	0.4748
miRNA-alternative	5	miRNA-null	21	5	21	0	0.026992	0.497
miRNA-alternative	5	miRNA-null	22	5	22	0	3.88E-05	0.4352
miRNA-alternative	5	miRNA-null	23	5	23	0	0.006042	0.446
miRNA-alternative	5	miRNA-null	24	5	24	0	0.008115	0.469066667
miRNA-alternative	5	miRNA-null	25	5	25	0	0.019773	0.4746
miRNA-alternative	5	miRNA-null	26	5	26	0	0.022787	0.477733333
miRNA-alternative	5	miRNA-null	27	5	27	0	0.028588	0.478733333
miRNA-alternative	5	miRNA-null	28	5	28	0	0.015845	0.472333333
miRNA-alternative	5	miRNA-null	29	5	29	0	0.022927	0.467466667
miRNA-alternative	5	miRNA-null	30	5	30	0	0.028518	0.476466667
miRNA-alternative	5	miRNA-null	31	5	31	0	0.005434	0.474133333
miRNA-alternative	5	miRNA-null	32	5	32	0	0.028167	0.490933333
miRNA-alternative	5	miRNA-null	33	5	33	0	0.004639	0.458133333
miRNA-alternative	5	miRNA-null	34	5	34	0	0.021699	0.4754
miRNA-alternative	5	miRNA-null	35	5	35	0	0.01531	0.451666667
miRNA-alternative	5	miRNA-null	36	5	36	0	0.022377	0.470666667
miRNA-alternative	5	miRNA-null	37	5	37	0	0.027085	0.478866667
miRNA-alternative	5	miRNA-null	38	5	38	0	0.021879	0.475866667
miRNA-alternative	5	miRNA-null	39	5	39	0	0.028499	0.481666667
miRNA-alternative	5	miRNA-null	40	5	40	0	0.027069	0.476066667
miRNA-alternative	6	miRNA-null	7	6	7	0	0.274832	0.510333333
miRNA-alternative	6	miRNA-null	8	6	8	0	0.003337	0.450466667
miRNA-alternative	6	miRNA-null	9	6	9	0	0.11931	0.4804
miRNA-alternative	6	miRNA-null	10	6	10	0	0.01825	0.4628
miRNA-alternative	6	miRNA-null	11	6	11	0	0.063721	0.481333333
miRNA-alternative	6	miRNA-null	12	6	12	0	0.406504	0.514466667
miRNA-alternative	6	miRNA-null	13	6	13	0	0.471776	0.519133333
miRNA-alternative	6	miRNA-null	14	6	14	0	0.439443	0.522933333
miRNA-alternative	6	miRNA-null	15	6	15	0	0.021842	0.4698
miRNA-alternative	6	miRNA-null	16	6	16	0	0.321417	0.503533333
miRNA-alternative	6	miRNA-null	17	6	17	0	0.400746	0.509866667
miRNA-alternative	6	miRNA-null	18	6	18	0	0.435312	0.516866667
miRNA-alternative	6	miRNA-null	19	6	19	0	0.032698	0.469533333
miRNA-alternative	6	miRNA-null	20	6	20	0	0.240899	0.5108
miRNA-alternative	6	miRNA-null	21	6	21	0	0.449188	0.5186
miRNA-alternative	6	miRNA-null	22	6	22	0	0.000255	0.443266667
miRNA-alternative	6	miRNA-null	23	6	23	0	0.064429	0.464866667
miRNA-alternative	6	miRNA-null	24	6	24	0	0.110736	0.488466667
miRNA-alternative	6	miRNA-null	25	6	25	0	0.326478	0.513133333
miRNA-alternative	6	miRNA-null	26	6	26	0	0.342824	0.497266667
miRNA-alternative	6	miRNA-null	27	6	27	0	0.487527	0.5292
miRNA-alternative	6	miRNA-null	28	6	28	0	0.205731	0.492133333
miRNA-alternative	6	miRNA-null	29	6	29	0	0.386558	0.505333333
miRNA-alternative	6	miRNA-null	30	6	30	0	0.488963	0.521333333
miRNA-alternative	6	miRNA-null	31	6	31	0	0.07889	0.489066667
miRNA-alternative	6	miRNA-null	32	6	32	0	0.482322	0.5214
miRNA-alternative	6	miRNA-null	33	6	33	0	0.059697	0.481866667
miRNA-alternative	6	miRNA-null	34	6	34	0	0.329807	0.5064
miRNA-alternative	6	miRNA-null	35	6	35	0	0.214776	0.494866667

miRNA-alternative 6	miRNA-null 36	6	36	0	0.314031	0.5096
miRNA-alternative 6	miRNA-null 37	6	37	0	0.44431	0.516466667
miRNA-alternative 6	miRNA-null 38	6	38	0	0.325403	0.501133333
miRNA-alternative 6	miRNA-null 39	6	39	0	0.488196	0.522666667
miRNA-alternative 6	miRNA-null 40	6	40	0	0.455779	0.516
miRNA-null 7	miRNA-null 7	7	8	0	0.002458	0.458866667
miRNA-null 7	miRNA-null 7	7	9	0	0.104197	0.4834
miRNA-null 7	miRNA-null 7	7	10	0	0.017966	0.4678
miRNA-null 7	miRNA-null 7	7	11	0	0.048182	0.486133333
miRNA-null 7	miRNA-null 7	7	12	0	0.326955	0.522533333
miRNA-null 7	miRNA-null 7	7	13	0	0.372011	0.514133333
miRNA-null 7	miRNA-null 7	7	14	0	0.355097	0.521466667
miRNA-null 7	miRNA-null 7	7	15	0	0.015407	0.483533333
miRNA-null 7	miRNA-null 7	7	16	0	0.27361	0.505266667
miRNA-null 7	miRNA-null 7	7	17	0	0.302921	0.510333333
miRNA-null 7	miRNA-null 7	7	18	0	0.356406	0.526266667
miRNA-null 7	miRNA-null 7	7	19	0	0.02078	0.468533333
miRNA-null 7	miRNA-null 7	7	20	0	0.183852	0.498133333
miRNA-null 7	miRNA-null 7	7	21	0	0.352694	0.5272
miRNA-null 7	miRNA-null 7	7	22	0	0.000214	0.4362
miRNA-null 7	miRNA-null 7	7	23	0	0.058991	0.4634
miRNA-null 7	miRNA-null 7	7	24	0	0.09487	0.505666667
miRNA-null 7	miRNA-null 7	7	25	0	0.274242	0.509266667
miRNA-null 7	miRNA-null 7	7	26	0	0.28209	0.502133333
miRNA-null 7	miRNA-null 7	7	27	0	0.37739	0.525933333
miRNA-null 7	miRNA-null 7	7	28	0	0.163743	0.493133333
miRNA-null 7	miRNA-null 7	7	29	0	0.336563	0.520733333
miRNA-null 7	miRNA-null 7	7	30	0	0.385413	0.531133333
miRNA-null 7	miRNA-null 7	7	31	0	0.079907	0.488666667
miRNA-null 7	miRNA-null 7	7	32	0	0.379768	0.518533333
miRNA-null 7	miRNA-null 7	7	33	0	0.055057	0.5
miRNA-null 7	miRNA-null 7	7	34	0	0.253211	0.5182
miRNA-null 7	miRNA-null 7	7	35	0	0.171023	0.5166
miRNA-null 7	miRNA-null 7	7	36	0	0.240272	0.512266667
miRNA-null 7	miRNA-null 7	7	37	0	0.350616	0.5176
miRNA-null 7	miRNA-null 7	7	38	0	0.277759	0.511066667
miRNA-null 7	miRNA-null 7	7	39	0	0.384899	0.516266667
miRNA-null 7	miRNA-null 7	7	40	0	0.367256	0.522466667
miRNA-null 8	miRNA-null 8	8	9	0	0.001989	0.453733333
miRNA-null 8	miRNA-null 8	8	10	0	0.000263	0.456
miRNA-null 8	miRNA-null 8	8	11	0	0.000734	0.455466667
miRNA-null 8	miRNA-null 8	8	12	0	0.003635	0.4596
miRNA-null 8	miRNA-null 8	8	13	0	0.003772	0.443933333
miRNA-null 8	miRNA-null 8	8	14	0	0.003884	0.4608
miRNA-null 8	miRNA-null 8	8	15	0	0.000448	0.433733333
miRNA-null 8	miRNA-null 8	8	16	0	0.003678	0.455533333
miRNA-null 8	miRNA-null 8	8	17	0	0.003666	0.456733333
miRNA-null 8	miRNA-null 8	8	18	0	0.00379	0.4578
miRNA-null 8	miRNA-null 8	8	19	0	0.000286	0.437866667
miRNA-null 8	miRNA-null 8	8	20	0	0.002235	0.423733333
miRNA-null 8	miRNA-null 8	8	21	0	0.003903	0.454933333
miRNA-null 8	miRNA-null 8	8	22	0	4.68E-06	0.4282
miRNA-null 8	miRNA-null 8	8	23	0	0.0011	0.436
miRNA-null 8	miRNA-null 8	8	24	0	0.001004	0.443133333

miRNA-null 8	miRNA-null 8	8	25	0	0.003228	0.455466667
miRNA-null 8	miRNA-null 8	8	26	0	0.002866	0.451733333
miRNA-null 8	miRNA-null 8	8	27	0	0.003884	0.455733333
miRNA-null 8	miRNA-null 8	8	28	0	0.0024	0.453533333
miRNA-null 8	miRNA-null 8	8	29	0	0.00346	0.461666667
miRNA-null 8	miRNA-null 8	8	30	0	0.004079	0.465733333
miRNA-null 8	miRNA-null 8	8	31	0	0.001201	0.455466667
miRNA-null 8	miRNA-null 8	8	32	0	0.004013	0.462133333
miRNA-null 8	miRNA-null 8	8	33	0	0.000873	0.4396
miRNA-null 8	miRNA-null 8	8	34	0	0.003487	0.446666667
miRNA-null 8	miRNA-null 8	8	35	0	0.002005	0.457533333
miRNA-null 8	miRNA-null 8	8	36	0	0.003183	0.456533333
miRNA-null 8	miRNA-null 8	8	37	0	0.003895	0.452
miRNA-null 8	miRNA-null 8	8	38	0	0.002577	0.460666667
miRNA-null 8	miRNA-null 8	8	39	0	0.003988	0.461333333
miRNA-null 8	miRNA-null 8	8	40	0	0.003795	0.462866667
miRNA-null 9	miRNA-null 9	9	10	0	0.009767	0.469466667
miRNA-null 9	miRNA-null 9	9	11	0	0.029327	0.4746
miRNA-null 9	miRNA-null 9	9	12	0	0.156696	0.4742
miRNA-null 9	miRNA-null 9	9	13	0	0.162284	0.4796
miRNA-null 9	miRNA-null 9	9	14	0	0.161768	0.484466667
miRNA-null 9	miRNA-null 9	9	15	0	0.009325	0.466133333
miRNA-null 9	miRNA-null 9	9	16	0	0.132083	0.468333333
miRNA-null 9	miRNA-null 9	9	17	0	0.137759	0.485466667
miRNA-null 9	miRNA-null 9	9	18	0	0.154975	0.496933333
miRNA-null 9	miRNA-null 9	9	19	0	0.013521	0.462066667
miRNA-null 9	miRNA-null 9	9	20	0	0.092025	0.4856
miRNA-null 9	miRNA-null 9	9	21	0	0.152231	0.477
miRNA-null 9	miRNA-null 9	9	22	0	0.000136	0.425733333
miRNA-null 9	miRNA-null 9	9	23	0	0.023996	0.459666667
miRNA-null 9	miRNA-null 9	9	24	0	0.053375	0.482066667
miRNA-null 9	miRNA-null 9	9	25	0	0.139574	0.482933333
miRNA-null 9	miRNA-null 9	9	26	0	0.122999	0.468866667
miRNA-null 9	miRNA-null 9	9	27	0	0.168821	0.492133333
miRNA-null 9	miRNA-null 9	9	28	0	0.083114	0.461933333
miRNA-null 9	miRNA-null 9	9	29	0	0.14678	0.4778
miRNA-null 9	miRNA-null 9	9	30	0	0.168409	0.487466667
miRNA-null 9	miRNA-null 9	9	31	0	0.03636	0.4642
miRNA-null 9	miRNA-null 9	9	32	0	0.169131	0.487
miRNA-null 9	miRNA-null 9	9	33	0	0.029478	0.4648
miRNA-null 9	miRNA-null 9	9	34	0	0.1242	0.474933333
miRNA-null 9	miRNA-null 9	9	35	0	0.083735	0.4698
miRNA-null 9	miRNA-null 9	9	36	0	0.120686	0.486866667
miRNA-null 9	miRNA-null 9	9	37	0	0.162236	0.481733333
miRNA-null 9	miRNA-null 9	9	38	0	0.111109	0.4774
miRNA-null 9	miRNA-null 9	9	39	0	0.16922	0.4854
miRNA-null 9	miRNA-null 9	9	40	0	0.163428	0.4886
miRNA-null 10	miRNA-null 10	10	11	0	0.006145	0.4418
miRNA-null 10	miRNA-null 10	10	12	0	0.023603	0.462733333
miRNA-null 10	miRNA-null 10	10	13	0	0.02467	0.470266667
miRNA-null 10	miRNA-null 10	10	14	0	0.02249	0.455533333
miRNA-null 10	miRNA-null 10	10	15	0	0.002394	0.469666667
miRNA-null 10	miRNA-null 10	10	16	0	0.019531	0.466066667
miRNA-null 10	miRNA-null 10	10	17	0	0.021379	0.4682

miRNA-null 10	miRNA-null 10	10	18	0	0.023391	0.468266667
miRNA-null 10	miRNA-null 10	10	19	0	0.002316	0.443466667
miRNA-null 10	miRNA-null 10	10	20	0	0.01385	0.479466667
miRNA-null 10	miRNA-null 10	10	21	0	0.022164	0.4662
miRNA-null 10	miRNA-null 10	10	22	0	2.15E-05	0.4118
miRNA-null 10	miRNA-null 10	10	23	0	0.006722	0.4432
miRNA-null 10	miRNA-null 10	10	24	0	0.00741	0.4618
miRNA-null 10	miRNA-null 10	10	25	0	0.020507	0.470933333
miRNA-null 10	miRNA-null 10	10	26	0	0.019531	0.471733333
miRNA-null 10	miRNA-null 10	10	27	0	0.024369	0.467733333
miRNA-null 10	miRNA-null 10	10	28	0	0.012224	0.472266667
miRNA-null 10	miRNA-null 10	10	29	0	0.021882	0.4664
miRNA-null 10	miRNA-null 10	10	30	0	0.025086	0.4708
miRNA-null 10	miRNA-null 10	10	31	0	0.004737	0.449266667
miRNA-null 10	miRNA-null 10	10	32	0	0.02505	0.475133333
miRNA-null 10	miRNA-null 10	10	33	0	0.004976	0.4714
miRNA-null 10	miRNA-null 10	10	34	0	0.01923	0.4698
miRNA-null 10	miRNA-null 10	10	35	0	0.014827	0.461933333
miRNA-null 10	miRNA-null 10	10	36	0	0.020887	0.465533333
miRNA-null 10	miRNA-null 10	10	37	0	0.023678	0.4748
miRNA-null 10	miRNA-null 10	10	38	0	0.020961	0.4656
miRNA-null 10	miRNA-null 10	10	39	0	0.025067	0.471266667
miRNA-null 10	miRNA-null 10	10	40	0	0.023984	0.462933333
miRNA-null 11	miRNA-null 11	11	12	0	0.080572	0.480666667
miRNA-null 11	miRNA-null 11	11	13	0	0.07874	0.474266667
miRNA-null 11	miRNA-null 11	11	14	0	0.07662	0.481133333
miRNA-null 11	miRNA-null 11	11	15	0	0.004512	0.457466667
miRNA-null 11	miRNA-null 11	11	16	0	0.061969	0.482466667
miRNA-null 11	miRNA-null 11	11	17	0	0.068602	0.4706
miRNA-null 11	miRNA-null 11	11	18	0	0.0773	0.484
miRNA-null 11	miRNA-null 11	11	19	0	0.008707	0.474
miRNA-null 11	miRNA-null 11	11	20	0	0.045783	0.483933333
miRNA-null 11	miRNA-null 11	11	21	0	0.076251	0.475
miRNA-null 11	miRNA-null 11	11	22	0	4.91E-05	0.445466667
miRNA-null 11	miRNA-null 11	11	23	0	0.012285	0.456266667
miRNA-null 11	miRNA-null 11	11	24	0	0.02604	0.475333333
miRNA-null 11	miRNA-null 11	11	25	0	0.058182	0.485933333
miRNA-null 11	miRNA-null 11	11	26	0	0.062412	0.457466667
miRNA-null 11	miRNA-null 11	11	27	0	0.081678	0.484133333
miRNA-null 11	miRNA-null 11	11	28	0	0.03655	0.470733333
miRNA-null 11	miRNA-null 11	11	29	0	0.074209	0.484066667
miRNA-null 11	miRNA-null 11	11	30	0	0.083434	0.4892
miRNA-null 11	miRNA-null 11	11	31	0	0.020293	0.4674
miRNA-null 11	miRNA-null 11	11	32	0	0.083128	0.474466667
miRNA-null 11	miRNA-null 11	11	33	0	0.014633	0.448533333
miRNA-null 11	miRNA-null 11	11	34	0	0.072192	0.484666667
miRNA-null 11	miRNA-null 11	11	35	0	0.049728	0.477466667
miRNA-null 11	miRNA-null 11	11	36	0	0.059167	0.475266667
miRNA-null 11	miRNA-null 11	11	37	0	0.074932	0.484
miRNA-null 11	miRNA-null 11	11	38	0	0.05816	0.491066667
miRNA-null 11	miRNA-null 11	11	39	0	0.083617	0.482866667
miRNA-null 11	miRNA-null 11	11	40	0	0.083269	0.478533333
miRNA-null 12	miRNA-null 12	12	13	0	0.643033	0.531333333
miRNA-null 12	miRNA-null 12	12	14	0	0.593867	0.527066667

miRNA-null 12	miRNA-null 12	12	15	0	0.026442	0.472533333
miRNA-null 12	miRNA-null 12	12	16	0	0.443375	0.5248
miRNA-null 12	miRNA-null 12	12	17	0	0.502599	0.521733333
miRNA-null 12	miRNA-null 12	12	18	0	0.59253	0.531533333
miRNA-null 12	miRNA-null 12	12	19	0	0.039394	0.472666667
miRNA-null 12	miRNA-null 12	12	20	0	0.262488	0.505666667
miRNA-null 12	miRNA-null 12	12	21	0	0.611764	0.532066667
miRNA-null 12	miRNA-null 12	12	22	0	0.000253	0.4576
miRNA-null 12	miRNA-null 12	12	23	0	0.078976	0.4696
miRNA-null 12	miRNA-null 12	12	24	0	0.133173	0.4886
miRNA-null 12	miRNA-null 12	12	25	0	0.437951	0.517733333
miRNA-null 12	miRNA-null 12	12	26	0	0.404725	0.510133333
miRNA-null 12	miRNA-null 12	12	27	0	0.672763	0.537866667
miRNA-null 12	miRNA-null 12	12	28	0	0.25634	0.501666667
miRNA-null 12	miRNA-null 12	12	29	0	0.521225	0.5182
miRNA-null 12	miRNA-null 12	12	30	0	0.684392	0.5356
miRNA-null 12	miRNA-null 12	12	31	0	0.106017	0.489066667
miRNA-null 12	miRNA-null 12	12	32	0	0.676628	0.532533333
miRNA-null 12	miRNA-null 12	12	33	0	0.077691	0.483133333
miRNA-null 12	miRNA-null 12	12	34	0	0.423513	0.499733333
miRNA-null 12	miRNA-null 12	12	35	0	0.270127	0.5076
miRNA-null 12	miRNA-null 12	12	36	0	0.387834	0.5194
miRNA-null 12	miRNA-null 12	12	37	0	0.606323	0.524
miRNA-null 12	miRNA-null 12	12	38	0	0.414855	0.5164
miRNA-null 12	miRNA-null 12	12	39	0	0.681319	0.527533333
miRNA-null 12	miRNA-null 12	12	40	0	0.641208	0.529266667
miRNA-null 13	miRNA-null 13	13	14	0	0.676764	0.525333333
miRNA-null 13	miRNA-null 13	13	15	0	0.027703	0.477466667
miRNA-null 13	miRNA-null 13	13	16	0	0.474931	0.526
miRNA-null 13	miRNA-null 13	13	17	0	0.555433	0.526266667
miRNA-null 13	miRNA-null 13	13	18	0	0.675795	0.530466667
miRNA-null 13	miRNA-null 13	13	19	0	0.039516	0.4746
miRNA-null 13	miRNA-null 13	13	20	0	0.310073	0.5006
miRNA-null 13	miRNA-null 13	13	21	0	0.714045	0.5344
miRNA-null 13	miRNA-null 13	13	22	0	0.000269	0.443733333
miRNA-null 13	miRNA-null 13	13	23	0	0.082849	0.4748
miRNA-null 13	miRNA-null 13	13	24	0	0.146895	0.494866667
miRNA-null 13	miRNA-null 13	13	25	0	0.487106	0.522266667
miRNA-null 13	miRNA-null 13	13	26	0	0.492531	0.511533333
miRNA-null 13	miRNA-null 13	13	27	0	0.799982	0.533333333
miRNA-null 13	miRNA-null 13	13	28	0	0.27219	0.489266667
miRNA-null 13	miRNA-null 13	13	29	0	0.610071	0.524133333
miRNA-null 13	miRNA-null 13	13	30	0	0.823749	0.535866667
miRNA-null 13	miRNA-null 13	13	31	0	0.112611	0.4876
miRNA-null 13	miRNA-null 13	13	32	0	0.806817	0.533133333
miRNA-null 13	miRNA-null 13	13	33	0	0.084382	0.4912
miRNA-null 13	miRNA-null 13	13	34	0	0.465549	0.513466667
miRNA-null 13	miRNA-null 13	13	35	0	0.289895	0.510466667
miRNA-null 13	miRNA-null 13	13	36	0	0.425648	0.518466667
miRNA-null 13	miRNA-null 13	13	37	0	0.683765	0.534266667
miRNA-null 13	miRNA-null 13	13	38	0	0.444616	0.509733333
miRNA-null 13	miRNA-null 13	13	39	0	0.815463	0.532
miRNA-null 13	miRNA-null 13	13	40	0	0.73403	0.526666667
miRNA-null 14	miRNA-null 14	14	15	0	0.026728	0.475666667

miRNA-null 14	miRNA-null 14	14	16	0	0.441759	0.522
miRNA-null 14	miRNA-null 14	14	17	0	0.511451	0.5262
miRNA-null 14	miRNA-null 14	14	18	0	0.618261	0.5312
miRNA-null 14	miRNA-null 14	14	19	0	0.034856	0.471266667
miRNA-null 14	miRNA-null 14	14	20	0	0.283973	0.5138
miRNA-null 14	miRNA-null 14	14	21	0	0.647258	0.5288
miRNA-null 14	miRNA-null 14	14	22	0	0.000273	0.453533333
miRNA-null 14	miRNA-null 14	14	23	0	0.076723	0.477266667
miRNA-null 14	miRNA-null 14	14	24	0	0.151406	0.490933333
miRNA-null 14	miRNA-null 14	14	25	0	0.46305	0.519266667
miRNA-null 14	miRNA-null 14	14	26	0	0.451299	0.515533333
miRNA-null 14	miRNA-null 14	14	27	0	0.709483	0.5354
miRNA-null 14	miRNA-null 14	14	28	0	0.25627	0.497266667
miRNA-null 14	miRNA-null 14	14	29	0	0.547916	0.5206
miRNA-null 14	miRNA-null 14	14	30	0	0.723442	0.532733333
miRNA-null 14	miRNA-null 14	14	31	0	0.110434	0.494933333
miRNA-null 14	miRNA-null 14	14	32	0	0.709964	0.526933333
miRNA-null 14	miRNA-null 14	14	33	0	0.081897	0.489733333
miRNA-null 14	miRNA-null 14	14	34	0	0.440239	0.521666667
miRNA-null 14	miRNA-null 14	14	35	0	0.276778	0.496933333
miRNA-null 14	miRNA-null 14	14	36	0	0.404449	0.520666667
miRNA-null 14	miRNA-null 14	14	37	0	0.629736	0.5238
miRNA-null 14	miRNA-null 14	14	38	0	0.441795	0.5152
miRNA-null 14	miRNA-null 14	14	39	0	0.716404	0.532066667
miRNA-null 14	miRNA-null 14	14	40	0	0.665086	0.535
miRNA-null 15	miRNA-null 15	15	16	0	0.023074	0.469733333
miRNA-null 15	miRNA-null 15	15	17	0	0.024911	0.4706
miRNA-null 15	miRNA-null 15	15	18	0	0.025012	0.4786
miRNA-null 15	miRNA-null 15	15	19	0	0.002865	0.453666667
miRNA-null 15	miRNA-null 15	15	20	0	0.017095	0.477533333
miRNA-null 15	miRNA-null 15	15	21	0	0.026259	0.4786
miRNA-null 15	miRNA-null 15	15	22	0	2.83E-05	0.446533333
miRNA-null 15	miRNA-null 15	15	23	0	0.004142	0.445866667
miRNA-null 15	miRNA-null 15	15	24	0	0.008944	0.468133333
miRNA-null 15	miRNA-null 15	15	25	0	0.022027	0.4684
miRNA-null 15	miRNA-null 15	15	26	0	0.020075	0.476466667
miRNA-null 15	miRNA-null 15	15	27	0	0.027784	0.4688
miRNA-null 15	miRNA-null 15	15	28	0	0.017201	0.465466667
miRNA-null 15	miRNA-null 15	15	29	0	0.024791	0.474
miRNA-null 15	miRNA-null 15	15	30	0	0.027927	0.474533333
miRNA-null 15	miRNA-null 15	15	31	0	0.006239	0.477066667
miRNA-null 15	miRNA-null 15	15	32	0	0.027759	0.481933333
miRNA-null 15	miRNA-null 15	15	33	0	0.003789	0.457266667
miRNA-null 15	miRNA-null 15	15	34	0	0.023115	0.461866667
miRNA-null 15	miRNA-null 15	15	35	0	0.01484	0.4698
miRNA-null 15	miRNA-null 15	15	36	0	0.017623	0.482066667
miRNA-null 15	miRNA-null 15	15	37	0	0.026711	0.4842
miRNA-null 15	miRNA-null 15	15	38	0	0.020755	0.478266667
miRNA-null 15	miRNA-null 15	15	39	0	0.027741	0.472333333
miRNA-null 15	miRNA-null 15	15	40	0	0.026221	0.479266667
miRNA-null 16	miRNA-null 16	16	17	0	0.3774	0.5186
miRNA-null 16	miRNA-null 16	16	18	0	0.45404	0.525866667
miRNA-null 16	miRNA-null 16	16	19	0	0.032173	0.4728
miRNA-null 16	miRNA-null 16	16	20	0	0.231635	0.526333333

miRNA-null 16	miRNA-null 16	16	21	0	0.465534	0.523666667
miRNA-null 16	miRNA-null 16	16	22	0	0.000253	0.448733333
miRNA-null 16	miRNA-null 16	16	23	0	0.059556	0.452333333
miRNA-null 16	miRNA-null 16	16	24	0	0.115438	0.506866667
miRNA-null 16	miRNA-null 16	16	25	0	0.346379	0.523866667
miRNA-null 16	miRNA-null 16	16	26	0	0.345245	0.510666667
miRNA-null 16	miRNA-null 16	16	27	0	0.500874	0.5304
miRNA-null 16	miRNA-null 16	16	28	0	0.219854	0.493
miRNA-null 16	miRNA-null 16	16	29	0	0.394579	0.523866667
miRNA-null 16	miRNA-null 16	16	30	0	0.50554	0.526466667
miRNA-null 16	miRNA-null 16	16	31	0	0.081122	0.489066667
miRNA-null 16	miRNA-null 16	16	32	0	0.500967	0.526533333
miRNA-null 16	miRNA-null 16	16	33	0	0.064506	0.484866667
miRNA-null 16	miRNA-null 16	16	34	0	0.323656	0.509133333
miRNA-null 16	miRNA-null 16	16	35	0	0.210536	0.492933333
miRNA-null 16	miRNA-null 16	16	36	0	0.304075	0.500133333
miRNA-null 16	miRNA-null 16	16	37	0	0.459778	0.525133333
miRNA-null 16	miRNA-null 16	16	38	0	0.319134	0.507133333
miRNA-null 16	miRNA-null 16	16	39	0	0.506442	0.527533333
miRNA-null 16	miRNA-null 16	16	40	0	0.470602	0.5356
miRNA-null 17	miRNA-null 17	17	18	0	0.521511	0.529933333
miRNA-null 17	miRNA-null 17	17	19	0	0.035282	0.473933333
miRNA-null 17	miRNA-null 17	17	20	0	0.267151	0.509133333
miRNA-null 17	miRNA-null 17	17	21	0	0.540836	0.512666667
miRNA-null 17	miRNA-null 17	17	22	0	0.000229	0.466733333
miRNA-null 17	miRNA-null 17	17	23	0	0.067405	0.471266667
miRNA-null 17	miRNA-null 17	17	24	0	0.115212	0.495266667
miRNA-null 17	miRNA-null 17	17	25	0	0.383225	0.506066667
miRNA-null 17	miRNA-null 17	17	26	0	0.390476	0.506333333
miRNA-null 17	miRNA-null 17	17	27	0	0.57646	0.522933333
miRNA-null 17	miRNA-null 17	17	28	0	0.230394	0.489533333
miRNA-null 17	miRNA-null 17	17	29	0	0.484685	0.508933333
miRNA-null 17	miRNA-null 17	17	30	0	0.586315	0.519466667
miRNA-null 17	miRNA-null 17	17	31	0	0.097695	0.484
miRNA-null 17	miRNA-null 17	17	32	0	0.581435	0.520266667
miRNA-null 17	miRNA-null 17	17	33	0	0.075684	0.479466667
miRNA-null 17	miRNA-null 17	17	34	0	0.390464	0.515933333
miRNA-null 17	miRNA-null 17	17	35	0	0.253314	0.4922
miRNA-null 17	miRNA-null 17	17	36	0	0.34231	0.4948
miRNA-null 17	miRNA-null 17	17	37	0	0.517444	0.517066667
miRNA-null 17	miRNA-null 17	17	38	0	0.370886	0.505066667
miRNA-null 17	miRNA-null 17	17	39	0	0.585065	0.516333333
miRNA-null 17	miRNA-null 17	17	40	0	0.550566	0.525466667
miRNA-null 18	miRNA-null 18	18	19	0	0.037657	0.468066667
miRNA-null 18	miRNA-null 18	18	20	0	0.279054	0.503466667
miRNA-null 18	miRNA-null 18	18	21	0	0.632477	0.533466667
miRNA-null 18	miRNA-null 18	18	22	0	0.000297	0.4448
miRNA-null 18	miRNA-null 18	18	23	0	0.07625	0.470666667
miRNA-null 18	miRNA-null 18	18	24	0	0.142724	0.4864
miRNA-null 18	miRNA-null 18	18	25	0	0.466724	0.518866667
miRNA-null 18	miRNA-null 18	18	26	0	0.43426	0.5086
miRNA-null 18	miRNA-null 18	18	27	0	0.696895	0.532666667
miRNA-null 18	miRNA-null 18	18	28	0	0.246868	0.4928
miRNA-null 18	miRNA-null 18	18	29	0	0.540133	0.516266667

miRNA-null 18	miRNA-null 18	18	30	0	0.713209	0.535466667
miRNA-null 18	miRNA-null 18	18	31	0	0.106438	0.481
miRNA-null 18	miRNA-null 18	18	32	0	0.703398	0.5394
miRNA-null 18	miRNA-null 18	18	33	0	0.07743	0.474933333
miRNA-null 18	miRNA-null 18	18	34	0	0.41322	0.517266667
miRNA-null 18	miRNA-null 18	18	35	0	0.28597	0.499466667
miRNA-null 18	miRNA-null 18	18	36	0	0.376584	0.515533333
miRNA-null 18	miRNA-null 18	18	37	0	0.62054	0.5242
miRNA-null 18	miRNA-null 18	18	38	0	0.418487	0.498466667
miRNA-null 18	miRNA-null 18	18	39	0	0.71033	0.5316
miRNA-null 18	miRNA-null 18	18	40	0	0.656178	0.533866667
miRNA-null 19	miRNA-null 19	19	20	0	0.017913	0.4548
miRNA-null 19	miRNA-null 19	19	21	0	0.038915	0.4708
miRNA-null 19	miRNA-null 19	19	22	0	2.56E-05	0.4532
miRNA-null 19	miRNA-null 19	19	23	0	0.00814	0.449066667
miRNA-null 19	miRNA-null 19	19	24	0	0.010984	0.466933333
miRNA-null 19	miRNA-null 19	19	25	0	0.031657	0.476333333
miRNA-null 19	miRNA-null 19	19	26	0	0.029837	0.4772
miRNA-null 19	miRNA-null 19	19	27	0	0.038094	0.4816
miRNA-null 19	miRNA-null 19	19	28	0	0.02267	0.469333333
miRNA-null 19	miRNA-null 19	19	29	0	0.034559	0.480133333
miRNA-null 19	miRNA-null 19	19	30	0	0.039696	0.472133333
miRNA-null 19	miRNA-null 19	19	31	0	0.009243	0.454933333
miRNA-null 19	miRNA-null 19	19	32	0	0.039361	0.474133333
miRNA-null 19	miRNA-null 19	19	33	0	0.008126	0.465333333
miRNA-null 19	miRNA-null 19	19	34	0	0.031292	0.470066667
miRNA-null 19	miRNA-null 19	19	35	0	0.026026	0.481866667
miRNA-null 19	miRNA-null 19	19	36	0	0.030327	0.465666667
miRNA-null 19	miRNA-null 19	19	37	0	0.037797	0.469866667
miRNA-null 19	miRNA-null 19	19	38	0	0.026597	0.4546
miRNA-null 19	miRNA-null 19	19	39	0	0.039699	0.4788
miRNA-null 19	miRNA-null 19	19	40	0	0.03803	0.469533333
miRNA-null 20	miRNA-null 20	20	21	0	0.305865	0.514266667
miRNA-null 20	miRNA-null 20	20	22	0	0.000134	0.4322
miRNA-null 20	miRNA-null 20	20	23	0	0.041461	0.466333333
miRNA-null 20	miRNA-null 20	20	24	0	0.084082	0.509466667
miRNA-null 20	miRNA-null 20	20	25	0	0.237071	0.505066667
miRNA-null 20	miRNA-null 20	20	26	0	0.240294	0.505933333
miRNA-null 20	miRNA-null 20	20	27	0	0.320709	0.518866667
miRNA-null 20	miRNA-null 20	20	28	0	0.146099	0.490266667
miRNA-null 20	miRNA-null 20	20	29	0	0.271917	0.514866667
miRNA-null 20	miRNA-null 20	20	30	0	0.32061	0.519866667
miRNA-null 20	miRNA-null 20	20	31	0	0.068608	0.480133333
miRNA-null 20	miRNA-null 20	20	32	0	0.321386	0.516933333
miRNA-null 20	miRNA-null 20	20	33	0	0.045415	0.464466667
miRNA-null 20	miRNA-null 20	20	34	0	0.236897	0.498066667
miRNA-null 20	miRNA-null 20	20	35	0	0.151204	0.4924
miRNA-null 20	miRNA-null 20	20	36	0	0.211618	0.500333333
miRNA-null 20	miRNA-null 20	20	37	0	0.294527	0.509133333
miRNA-null 20	miRNA-null 20	20	38	0	0.224014	0.502066667
miRNA-null 20	miRNA-null 20	20	39	0	0.320675	0.5162
miRNA-null 20	miRNA-null 20	20	40	0	0.304565	0.5098
miRNA-null 21	miRNA-null 21	21	22	0	0.000268	0.449533333
miRNA-null 21	miRNA-null 21	21	23	0	0.078864	0.481733333

miRNA-null 21	miRNA-null 21	21	24	0	0.13423	0.4944
miRNA-null 21	miRNA-null 21	21	25	0	0.469099	0.521666667
miRNA-null 21	miRNA-null 21	21	26	0	0.458528	0.511933333
miRNA-null 21	miRNA-null 21	21	27	0	0.748442	0.534066667
miRNA-null 21	miRNA-null 21	21	28	0	0.269901	0.491066667
miRNA-null 21	miRNA-null 21	21	29	0	0.577806	0.521733333
miRNA-null 21	miRNA-null 21	21	30	0	0.768886	0.530733333
miRNA-null 21	miRNA-null 21	21	31	0	0.112793	0.497866667
miRNA-null 21	miRNA-null 21	21	32	0	0.745638	0.5328
miRNA-null 21	miRNA-null 21	21	33	0	0.083561	0.4958
miRNA-null 21	miRNA-null 21	21	34	0	0.437358	0.522133333
miRNA-null 21	miRNA-null 21	21	35	0	0.284045	0.500533333
miRNA-null 21	miRNA-null 21	21	36	0	0.408411	0.519466667
miRNA-null 21	miRNA-null 21	21	37	0	0.658989	0.521666667
miRNA-null 21	miRNA-null 21	21	38	0	0.43258	0.510333333
miRNA-null 21	miRNA-null 21	21	39	0	0.761676	0.527333333
miRNA-null 21	miRNA-null 21	21	40	0	0.703136	0.531666667
miRNA-null 22	miRNA-null 22	22	23	0	7.74E-05	0.4296
miRNA-null 22	miRNA-null 22	22	24	0	0.000156	0.4388
miRNA-null 22	miRNA-null 22	22	25	0	0.000254	0.450133333
miRNA-null 22	miRNA-null 22	22	26	0	0.000242	0.438666667
miRNA-null 22	miRNA-null 22	22	27	0	0.000295	0.4464
miRNA-null 22	miRNA-null 22	22	28	0	0.000169	0.446466667
miRNA-null 22	miRNA-null 22	22	29	0	0.000291	0.4474
miRNA-null 22	miRNA-null 22	22	30	0	0.000297	0.444133333
miRNA-null 22	miRNA-null 22	22	31	0	9.76E-05	0.429
miRNA-null 22	miRNA-null 22	22	32	0	0.000297	0.446666667
miRNA-null 22	miRNA-null 22	22	33	0	8.21E-05	0.424
miRNA-null 22	miRNA-null 22	22	34	0	0.000214	0.459666667
miRNA-null 22	miRNA-null 22	22	35	0	0.000186	0.446666667
miRNA-null 22	miRNA-null 22	22	36	0	0.000207	0.446066667
miRNA-null 22	miRNA-null 22	22	37	0	0.000293	0.432133333
miRNA-null 22	miRNA-null 22	22	38	0	0.000251	0.443466667
miRNA-null 22	miRNA-null 22	22	39	0	0.000293	0.4474
miRNA-null 22	miRNA-null 22	22	40	0	0.000274	0.455466667
miRNA-null 23	miRNA-null 23	23	24	0	0.024117	0.4612
miRNA-null 23	miRNA-null 23	23	25	0	0.069663	0.475
miRNA-null 23	miRNA-null 23	23	26	0	0.063167	0.469
miRNA-null 23	miRNA-null 23	23	27	0	0.081478	0.469133333
miRNA-null 23	miRNA-null 23	23	28	0	0.040367	0.467133333
miRNA-null 23	miRNA-null 23	23	29	0	0.071073	0.465
miRNA-null 23	miRNA-null 23	23	30	0	0.083547	0.4778
miRNA-null 23	miRNA-null 23	23	31	0	0.023372	0.468066667
miRNA-null 23	miRNA-null 23	23	32	0	0.078823	0.476533333
miRNA-null 23	miRNA-null 23	23	33	0	0.016904	0.470466667
miRNA-null 23	miRNA-null 23	23	34	0	0.063056	0.4702
miRNA-null 23	miRNA-null 23	23	35	0	0.047379	0.479133333
miRNA-null 23	miRNA-null 23	23	36	0	0.060651	0.465466667
miRNA-null 23	miRNA-null 23	23	37	0	0.078296	0.48
miRNA-null 23	miRNA-null 23	23	38	0	0.067195	0.4838
miRNA-null 23	miRNA-null 23	23	39	0	0.083664	0.4764
miRNA-null 23	miRNA-null 23	23	40	0	0.077889	0.482066667
miRNA-null 24	miRNA-null 24	24	25	0	0.113275	0.489066667
miRNA-null 24	miRNA-null 24	24	26	0	0.113931	0.486866667

miRNA-null 24	miRNA-null 24	24	27	0	0.153357	0.4976
miRNA-null 24	miRNA-null 24	24	28	0	0.084186	0.471066667
miRNA-null 24	miRNA-null 24	24	29	0	0.137266	0.500866667
miRNA-null 24	miRNA-null 24	24	30	0	0.155064	0.496
miRNA-null 24	miRNA-null 24	24	31	0	0.043034	0.4768
miRNA-null 24	miRNA-null 24	24	32	0	0.154993	0.491666667
miRNA-null 24	miRNA-null 24	24	33	0	0.024229	0.459533333
miRNA-null 24	miRNA-null 24	24	34	0	0.108253	0.490066667
miRNA-null 24	miRNA-null 24	24	35	0	0.086095	0.478266667
miRNA-null 24	miRNA-null 24	24	36	0	0.116329	0.4922
miRNA-null 24	miRNA-null 24	24	37	0	0.145986	0.491266667
miRNA-null 24	miRNA-null 24	24	38	0	0.128333	0.4838
miRNA-null 24	miRNA-null 24	24	39	0	0.153695	0.488733333
miRNA-null 24	miRNA-null 24	24	40	0	0.145206	0.4986
miRNA-null 25	miRNA-null 25	25	26	0	0.346731	0.501666667
miRNA-null 25	miRNA-null 25	25	27	0	0.510727	0.525133333
miRNA-null 25	miRNA-null 25	25	28	0	0.19462	0.4808
miRNA-null 25	miRNA-null 25	25	29	0	0.424629	0.501733333
miRNA-null 25	miRNA-null 25	25	30	0	0.512083	0.521
miRNA-null 25	miRNA-null 25	25	31	0	0.099676	0.4912
miRNA-null 25	miRNA-null 25	25	32	0	0.504648	0.5152
miRNA-null 25	miRNA-null 25	25	33	0	0.074435	0.4788
miRNA-null 25	miRNA-null 25	25	34	0	0.321164	0.5048
miRNA-null 25	miRNA-null 25	25	35	0	0.226544	0.491933333
miRNA-null 25	miRNA-null 25	25	36	0	0.323028	0.500333333
miRNA-null 25	miRNA-null 25	25	37	0	0.461578	0.511666667
miRNA-null 25	miRNA-null 25	25	38	0	0.335244	0.501733333
miRNA-null 25	miRNA-null 25	25	39	0	0.509998	0.5124
miRNA-null 25	miRNA-null 25	25	40	0	0.493355	0.511533333
miRNA-null 26	miRNA-null 26	26	27	0	0.502975	0.515933333
miRNA-null 26	miRNA-null 26	26	28	0	0.211374	0.485933333
miRNA-null 26	miRNA-null 26	26	29	0	0.41895	0.5112
miRNA-null 26	miRNA-null 26	26	30	0	0.508031	0.519466667
miRNA-null 26	miRNA-null 26	26	31	0	0.080963	0.483466667
miRNA-null 26	miRNA-null 26	26	32	0	0.504055	0.519666667
miRNA-null 26	miRNA-null 26	26	33	0	0.069023	0.482066667
miRNA-null 26	miRNA-null 26	26	34	0	0.319776	0.5078
miRNA-null 26	miRNA-null 26	26	35	0	0.222747	0.490266667
miRNA-null 26	miRNA-null 26	26	36	0	0.322632	0.506733333
miRNA-null 26	miRNA-null 26	26	37	0	0.456111	0.513866667
miRNA-null 26	miRNA-null 26	26	38	0	0.332817	0.494933333
miRNA-null 26	miRNA-null 26	26	39	0	0.507855	0.512133333
miRNA-null 26	miRNA-null 26	26	40	0	0.484305	0.521
miRNA-null 27	miRNA-null 27	27	28	0	0.279902	0.5004
miRNA-null 27	miRNA-null 27	27	29	0	0.623338	0.5254
miRNA-null 27	miRNA-null 27	27	30	0	0.903288	0.537133333
miRNA-null 27	miRNA-null 27	27	31	0	0.113336	0.486333333
miRNA-null 27	miRNA-null 27	27	32	0	0.870945	0.535066667
miRNA-null 27	miRNA-null 27	27	33	0	0.086152	0.4868
miRNA-null 27	miRNA-null 27	27	34	0	0.478082	0.5224
miRNA-null 27	miRNA-null 27	27	35	0	0.30389	0.507866667
miRNA-null 27	miRNA-null 27	27	36	0	0.437817	0.510333333
miRNA-null 27	miRNA-null 27	27	37	0	0.719147	0.5272
miRNA-null 27	miRNA-null 27	27	38	0	0.466055	0.519533333

miRNA-null 27	miRNA-null 27	27	39	0	0.894049	0.536666667
miRNA-null 27	miRNA-null 27	27	40	0	0.787596	0.531533333
miRNA-null 28	miRNA-null 28	28	29	0	0.240921	0.495
miRNA-null 28	miRNA-null 28	28	30	0	0.282826	0.501466667
miRNA-null 28	miRNA-null 28	28	31	0	0.051594	0.481066667
miRNA-null 28	miRNA-null 28	28	32	0	0.283093	0.495866667
miRNA-null 28	miRNA-null 28	28	33	0	0.043792	0.480933333
miRNA-null 28	miRNA-null 28	28	34	0	0.196673	0.492533333
miRNA-null 28	miRNA-null 28	28	35	0	0.134918	0.4934
miRNA-null 28	miRNA-null 28	28	36	0	0.189146	0.496866667
miRNA-null 28	miRNA-null 28	28	37	0	0.265869	0.494133333
miRNA-null 28	miRNA-null 28	28	38	0	0.208657	0.4946
miRNA-null 28	miRNA-null 28	28	39	0	0.282982	0.4974
miRNA-null 28	miRNA-null 28	28	40	0	0.266433	0.495866667
miRNA-null 29	miRNA-null 29	29	30	0	0.637387	0.515
miRNA-null 29	miRNA-null 29	29	31	0	0.10854	0.495066667
miRNA-null 29	miRNA-null 29	29	32	0	0.62725	0.529133333
miRNA-null 29	miRNA-null 29	29	33	0	0.078159	0.4948
miRNA-null 29	miRNA-null 29	29	34	0	0.396546	0.508666667
miRNA-null 29	miRNA-null 29	29	35	0	0.259139	0.504133333
miRNA-null 29	miRNA-null 29	29	36	0	0.374829	0.507533333
miRNA-null 29	miRNA-null 29	29	37	0	0.556116	0.5194
miRNA-null 29	miRNA-null 29	29	38	0	0.396403	0.504333333
miRNA-null 29	miRNA-null 29	29	39	0	0.636905	0.520333333
miRNA-null 29	miRNA-null 29	29	40	0	0.596734	0.525866667
miRNA-null 30	miRNA-null 30	30	31	0	0.118083	0.484666667
miRNA-null 30	miRNA-null 30	30	32	0	0.910954	0.535533333
miRNA-null 30	miRNA-null 30	30	33	0	0.086811	0.485333333
miRNA-null 30	miRNA-null 30	30	34	0	0.481568	0.522933333
miRNA-null 30	miRNA-null 30	30	35	0	0.308535	0.5164
miRNA-null 30	miRNA-null 30	30	36	0	0.439746	0.514933333
miRNA-null 30	miRNA-null 30	30	37	0	0.73417	0.530733333
miRNA-null 30	miRNA-null 30	30	38	0	0.46942	0.5096
miRNA-null 30	miRNA-null 30	30	39	0	0.947524	0.529933333
miRNA-null 30	miRNA-null 30	30	40	0	0.803366	0.535533333
miRNA-null 31	miRNA-null 31	31	32	0	0.117201	0.4928
miRNA-null 31	miRNA-null 31	31	33	0	0.02578	0.471466667
miRNA-null 31	miRNA-null 31	31	34	0	0.088095	0.484933333
miRNA-null 31	miRNA-null 31	31	35	0	0.073936	0.4844
miRNA-null 31	miRNA-null 31	31	36	0	0.084275	0.481466667
miRNA-null 31	miRNA-null 31	31	37	0	0.111035	0.485866667
miRNA-null 31	miRNA-null 31	31	38	0	0.089965	0.476266667
miRNA-null 31	miRNA-null 31	31	39	0	0.118331	0.4944
miRNA-null 31	miRNA-null 31	31	40	0	0.11023	0.486
miRNA-null 32	miRNA-null 32	32	33	0	0.084932	0.482933333
miRNA-null 32	miRNA-null 32	32	34	0	0.477031	0.521466667
miRNA-null 32	miRNA-null 32	32	35	0	0.30293	0.504066667
miRNA-null 32	miRNA-null 32	32	36	0	0.440276	0.518
miRNA-null 32	miRNA-null 32	32	37	0	0.722842	0.526733333
miRNA-null 32	miRNA-null 32	32	38	0	0.464683	0.518533333
miRNA-null 32	miRNA-null 32	32	39	0	0.899143	0.533
miRNA-null 32	miRNA-null 32	32	40	0	0.785589	0.530533333
miRNA-null 33	miRNA-null 33	33	34	0	0.061804	0.4954
miRNA-null 33	miRNA-null 33	33	35	0	0.045494	0.4742

miRNA-null 33	miRNA-null 33	33	36	0	0.065301	0.480666667
miRNA-null 33	miRNA-null 33	33	37	0	0.077978	0.472866667
miRNA-null 33	miRNA-null 33	33	38	0	0.068089	0.483266667
miRNA-null 33	miRNA-null 33	33	39	0	0.08664	0.481466667
miRNA-null 33	miRNA-null 33	33	40	0	0.085913	0.496333333
miRNA-null 34	miRNA-null 34	34	35	0	0.223057	0.5064
miRNA-null 34	miRNA-null 34	34	36	0	0.296152	0.508066667
miRNA-null 34	miRNA-null 34	34	37	0	0.423249	0.520266667
miRNA-null 34	miRNA-null 34	34	38	0	0.308646	0.492133333
miRNA-null 34	miRNA-null 34	34	39	0	0.481058	0.518066667
miRNA-null 34	miRNA-null 34	34	40	0	0.460417	0.5208
miRNA-null 35	miRNA-null 35	35	36	0	0.200442	0.495
miRNA-null 35	miRNA-null 35	35	37	0	0.277176	0.4938
miRNA-null 35	miRNA-null 35	35	38	0	0.214218	0.4894
miRNA-null 35	miRNA-null 35	35	39	0	0.308842	0.511466667
miRNA-null 35	miRNA-null 35	35	40	0	0.302278	0.505
miRNA-null 36	miRNA-null 36	36	37	0	0.410253	0.506133333
miRNA-null 36	miRNA-null 36	36	38	0	0.286553	0.489466667
miRNA-null 36	miRNA-null 36	36	39	0	0.441297	0.511533333
miRNA-null 36	miRNA-null 36	36	40	0	0.42076	0.519933333
miRNA-null 37	miRNA-null 37	37	38	0	0.424864	0.5072
miRNA-null 37	miRNA-null 37	37	39	0	0.730962	0.53
miRNA-null 37	miRNA-null 37	37	40	0	0.664803	0.529
miRNA-null 38	miRNA-null 38	38	39	0	0.469205	0.512066667
miRNA-null 38	miRNA-null 38	38	40	0	0.44956	0.514066667
miRNA-null 39	miRNA-null 39	39	40	0	0.801111	0.5384
